# First Data on *Ornithodoros moubata* Aquaporins: Structural, Phylogenetic and Immunogenic Characterisation as Vaccine Targets

**DOI:** 10.3390/pathogens11060694

**Published:** 2022-06-16

**Authors:** Ricardo Pérez-Sánchez, Ana Laura Cano-Argüelles, María González-Sánchez, Ana Oleaga

**Affiliations:** Parasitology Laboratory, Institute of Natural Resources and Agrobiology (IRNASA, CSIC), Cordel de Merinas, 40-52, 37008 Salamanca, Spain; ana.cano@irnasa.csic.es (A.L.C.-A.); m.g@csic.es (M.G.-S.); ana.oleaga@irnasa.csic.es (A.O.)

**Keywords:** soft ticks, *Ornithodoros moubata*, aquaporins, tick vaccines, antigenic peptides

## Abstract

*Ornithodoros moubata* transmits African swine fever and human relapsing fever in Africa. The elimination of *O. moubata* populations from anthropic environments is expected to improve the prevention and control of these diseases. Tick vaccines have emerged as a sustainable method for tick control, and tick aquaporins (AQPs) are promising targets for tick vaccines due to their vital functions, immunogenicity and ease of access by neutralising host antibodies. This study aimed at the systematic identification of the AQPs expressed by *O. moubata* (OmAQPs) and their characterisation as vaccine targets. Therefore, AQP coding sequences were recovered from available transcriptomic datasets, followed by PCR amplification, cloning, sequence verification and the analysis of the AQP protein structure and epitope exposure. Seven OmAQPs were identified and characterised: six were aquaglyceroporins, and one was a water-specific aquaporin. All of these were expressed in the salivary glands and midgut and only three in the coxal glands. Epitope exposure analysis identified three extracellular domains in each AQP, which concentrate overlapping B and T cell epitopes, making them interesting vaccine targets. Based on these domain sequences, a set of ten antigenic peptides was designed, which showed adequate properties to be produced and tested in pilot vaccine trials.

## 1. Introduction

Ticks are haematophagous arthropods that parasitise reptiles, birds and mammals worldwide. Tick infestations represent a severe global burden to human and animal health because they transmit a large number of pathogens, including viruses, bacteria, protozoa and helminths, which affect humans, pets, livestock and wildlife [1,2,3].

There are two main tick families, the Argasidae (soft ticks) and the Ixodidae (hard ticks), which differ in a range of structural, biological and ecological properties, and a monotypic third tick family, the Nuttalliellidae, whose sole species (*Nuttalliella namaqua*) shows features of both soft and hard ticks [4,5]. Most ixodids are exophilic ticks that shelter on the soil and vegetation and actively quest for hosts. Once attached, they feed for several days and ingest up to 100 times their body weight in blood. As the feeding progresses, ixodids concentrate the blood meal in the midgut, and the excess water is extracted from the haemolymph by the salivary glands and secreted back into the host with tick saliva. Once engorged, they drop off the host, returning to the soil, where the immatures moult to the next developmental stage, and the females oviposit and die. Conversely, argasids are typically endophilic/nidicolous ticks. They live inside the nests and burrows of their hosts as well as in domestic animal facilities and human houses. There, they find the microenvironmental conditions required for their development and have easy access to host blood. Most argasids are fast feeders that complete their meal in less than 1 h and ingest almost 10 times their body weight in blood. After concentrating the blood meal in the midgut, argasids use a pair of specific organs instead of salivary glands, the so-called coxal glands, to extract the excess water from haemolymph and excrete it to the outside as a colourless fluid; this occurs during or shortly after the completion of feeding. Once engorged, they detach off the host and moult or reproduce inside their sheltered harbourages. Adult argasids can feed and reproduce up to 10 times before they die and can survive for years without feeding [6,7,8,9].

The argasid tick *Ornithodoros moubata* is widespread throughout Central, South and East Africa. In the wild, it lives associated with warthogs and other hosts inhabiting burrows, but it also colonises anthropic environments, hiding inside human dwellings and domestic animal housing, particularly pig premises [8]. *O. moubata* is the main vector of the African swine fever (ASF) virus and the human relapsing fever (TBRF) agent *Borrelia duttoni*. In addition, it was recently identified as a competent vector for the Q fever agent *Coxiella burnetti* [10]. ASF is an acute haemorrhagic lethal disease of swine, for which there is no treatment or vaccine, which limits pig production and provokes significant economic losses in affected countries [11,12]. TBRF caused by *B. duttoni* is endemic in East African countries, showing prevalence rates of up to 6.4% and perinatal mortality rates as high as 436/1000 [13,14]. The presence of *O. moubata* in anthropic environments contributes to the persistence of ASF and TBRF in endemic areas and may facilitate their spread into neighbouring areas. Therefore, the elimination of synanthropic populations of *O. moubata* is expected to significantly improve the prevention and control of these diseases.

Anti-tick vaccines have shown to be an alternative, cost-effective and sustainable method for the control of tick infestations and tick-borne diseases, with advantages over chemical acaricide agents [15,16]. Success in tick vaccine development is largely dependent on the identification of tick antigens that are able to induce protective immune responses in the hosts. The search for tick-protective antigens can be approached by selecting candidate antigens that play essential functions for tick survival and reproduction [17]. Particularly interesting as vaccine targets are the molecules and biological processes that have been specifically evolved by ticks to adapt to their strict hematophagous lifestyle. Namely, the processes related to host attachment, blood ingestion and host defence modulation, which are carried out by salivary proteins, and the processes related to blood digestion, including nutrient transport and metabolism, iron and haem group management, detoxification and responses to oxidative stress, which are accomplished by proteins expressed in the midgut [18,19].

In the last few years, our team has focused substantial efforts on the identification of protective antigens for the development of an anti-*O. moubata* vaccine. With this aim, the transcriptome and proteome of *O. moubata* midgut and salivary glands were obtained and screened for the selection of vaccine target candidates [18,20,21,22,23]. Several of these salivary and intestinal candidates were tested in animal vaccine trials and provided 39–44% individual protective efficacies, which reached 60% when the same antigens were administered together as a multicomponent vaccine [5,24]. Despite these promising results, a fully protective vaccine against *O. moubata* has not been obtained, and research efforts are ongoing to identify new and more protective antigens. One of these protective antigens might be the aquaporin protein family.

Aquaporins (AQPs) are a superfamily of transmembrane proteins evolutionarily conserved from bacteria to mammals that form pores in cell membranes through which water and small neutral solutes, including glycerol and urea, can be transported. The structure of aquaporins is highly conserved across taxa, consisting of six transmembrane domains that are connected by three extracellular and two cytoplasmic loops. The sequence signature for the AQP superfamily is two highly conserved asparagine–proline–alanine (NPA) boxes located apart in the primary sequence, which interact with each other to form a pore when the protein folds and acquires its tertiary structure. The NPA boxes and sequence motifs around the pore, such as those forming the so-called aromatic arginine (ar/R) constriction, determine the pore size, charge and permeability and, in turn, the AQP classification. AQPs are divided into three subfamilies: (i) water-selective aquaporins (wAQP), most of which transport water selectively; and (ii) aquaglyceroporins (gAQP) and (iii) superaquaporins (sAQP), both of which transport water and small solutes with sAQPs typically localised inside the cell [25,26,27,28].

Tick AQP sequences are increasingly detected in tick genome and transcriptome sequencing projects. Currently, 93 nucleotide and 149 protein sequences from tick AQPs can be accessed in the NCBInr and Uniprot databases, respectively (visited on 26 April 2022). However, only a handful of tick AQPs have been studied in-depth, most of them in ixodid ticks. AQPs have been found in salivary glands, digestive tract, Malpighian tubules, synganglia and reproductive organs. AQPs participate in multiple physiological processes that are essential for ticks, including saliva production, feeding, blood meal concentration and regulation of the subsequent osmotic stress. This implies that about 75% of the ingested water and ions are returned to the host via tick saliva in ixodids or excreted outside via coxal glands in argasids [29,30,31].

Three-dimensional (3D) modelling and B-cell epitope prediction tools showed that tick AQPs expose B-cell epitopes in their extracellular loops [32,33,34,35]. Thus, the putative binding of host antibodies to B-cell epitopes on the extracellular domains of AQPs in tick gut, salivary glands and coxal glands could interfere with AQP function, resulting in impaired water balance and probably in reduced tick feeding, reproduction and survival. Accordingly, AQPs were considered promising targets for tick vaccine development [17,36,37], and several studies have already demonstrated the protective potential of a number of ixodid AQPs. Cattle vaccination with recombinant AQP1 from *Rhipicephalus microplus* reduced tick infestations by 68–75% [31]. Rabbit vaccination with two different recombinant forms of *Ixodes ricinus* AQP1 reduced the infestations by *I. ricinus* larvae by 32% and 80%, respectively [38]. More recently, vaccination with synthetic peptides derived from the extracellular domains of *R. microplus* AQP2 reduced by 25% the number of ticks feeding to repletion [35]. In contrast, argasid AQPs have been very poorly studied, so information on their sequence, structure, function, tissue expression and protective potential as vaccine targets is almost non-existent. There is only one recent article that has reported two AQP sequences found in the *Ornithodoros erraticus* mialome [34]. This study demonstrated that the vaccination of rabbits with synthetic peptides designed from the extracellular domains of these AQPs induced strong humoral responses and provided partial but significant protection against *O. erraticus* infestations. These findings support the proposal that AQPs from both ixodids and argasids might be candidate protective antigens for tick vaccines and encourage further studies with this protein family.

Accordingly, the objective of this work was to scrutinise the previously obtained transcriptomes of the salivary glands and midgut of *O. moubata* females in order to identify the AQP family members expressed by this tick and to assess their potential as protective antigens for tick vaccine development. With this aim, the AQP coding sequences found in the *O. moubata* transcriptomes were cloned and sequenced, and their translated amino acid sequences were analysed in silico to predict their structure and epitope exposure and to identify antigenic peptides that could be tested in future vaccine trials.

## 2. Results

### 2.1. AQP Transcripts Found in O. moubata

Up to 27 transcripts annotated as AQPs were recovered from the *O. moubata* midgut and salivary gland transcriptomes. Nine of them contained full-length ORFs for AQP proteins, and seven were unique, non-duplicated sequences since the salivary transcripts OM_7339 and OM_21119 were identical, respectively, to midgut transcripts ci|000124891 and ci|000114723 (Table S1). These seven unique AQP ORFs were listed in Table 1 and given simplified names.

Transcripts ci|000144090, OM_20812 and OM_22982 are annotated as orthologues to the XP_029845132.1 aquaporin-9 from *Ixodes scapularis*; hence, they were considered as isoforms of the same protein. Similarly, transcripts ci|000114723/OM_21119 and ci|000148315, which are both annotated as orthologues to the CAR66115.1 water-specific aquaporin from *Rhipicephalus sanguineus*, were also considered isoforms of the same protein. Transcript ci|000124891/OM_7339 is an orthologue to the XP_029833586.1 aquaporin AQPAe.a-like from *I. scapularis* and transcript ci|000113997 is an orthologue to the CAX48963.1 aquaglyceroporin from *R. sanguineus*.

### 2.2. Tissue Expression and Sequence Verification of O. moubata AQPs (OmAQPs)

**Figure 1:**
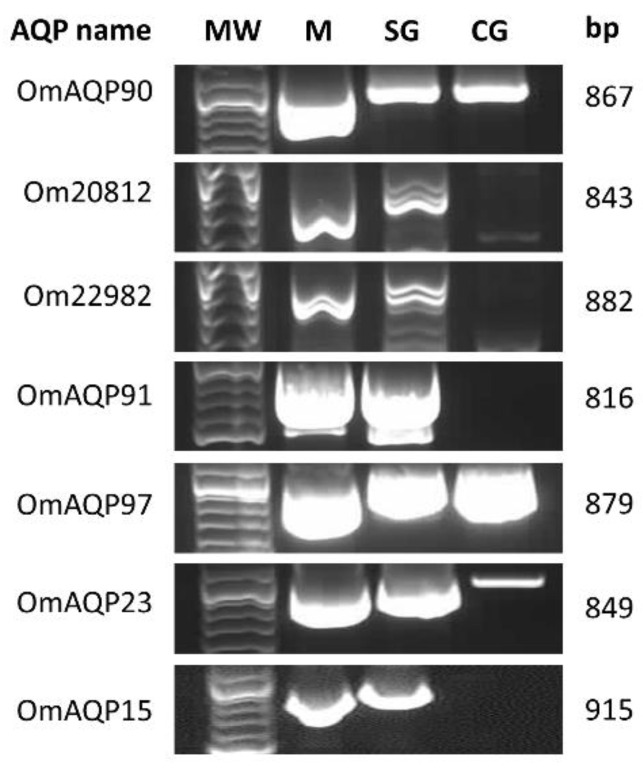
Agarose gel showing the PCR products from the amplification of AQP ORFs from *O. moubata* midgut (M), salivary glands (SG) and coxal glands (CG). MW, DNA ladder; bp, amplicon length in base pairs.

All seven AQP cDNA coding sequences were PCR-amplified from the salivary glands and midgut of *O. moubata*; three of them (ci|000144090, ci|000113997, ci|000114723/OM_21119) were also amplified from the *O. moubata* coxal glands, confirming their expression in these organs (Figure 1).

Cloning and sequencing of all these amplicons showed that, for every unique AQP, the sequences of the amplicons obtained from salivary glands, midgut and coxal glands were identical to each other and, in turn, identical to the sequence of the corresponding transcript. Only some single nucleotide positions varied between the sequences of amplicons and their homologous transcripts (Figure S1). This confirms the correct assembly and integrity of the AQP sequences, as previously obtained by RNA-seq [18,21]. 

The molecular characterisation of the predicted amino acid sequences of AQPs is shown in Table 2. Their lengths ranged from 271 to 304 amino acids, their MW from 28 to 33 kDa and their pI ranged from acidic values to values around neutrality (5.1–7.7). All OmAQPs lacked signal peptides, GPI anchor sites and N-glycosylation motifs. As proteins lacking signal peptides are unlikely to be exposed to the N-glycosylation machinery, they may not be glycosylated, even though they contain potential motifs. Two O-glycosylation sites were predicted for OmAQP90, Om20812 and Om22982 isoforms: namely, on residues threonine 128 and serine 132 for OmAQP90 and on residues threonine 126 and 128 for Om20812 and Om22982. Finally, six out of seven OmAQPs were predicted as probable antigens (VaxiJen score above 0.5), and only OmAQP91 was predicted as a probable non-antigen (VaxiJen score below 0.5).

### 2.3. Phylogenetic Analysis

Searches of the Uniprot and NCBInr databases for orthologues of OmAQPs in ticks retrieved between 5 and 14 top matches meeting the selection criteria (Table S2).

The OmAQP90, Om20812 and Om22982 isoforms matched to 11 orthologues each, most of which were redundant as they matched simultaneously to two or three isoforms. The elimination of redundancies left 13 unique matches, comprising 1 argasid and 12 ixodid AQP sequences. The OmAQP23 and OmAQP15 isoforms matched to the same top 14 orthologues, comprising 4 argasid and 10 ixodid AQP sequences. OmAQP97 matched to 10 orthologues, among which two were from argasids and eight from ixodids. Finally, OmAQP91 matched to only five orthologues: one argasid and four ixodid AQPs (Table S2).

**Figure 2:**
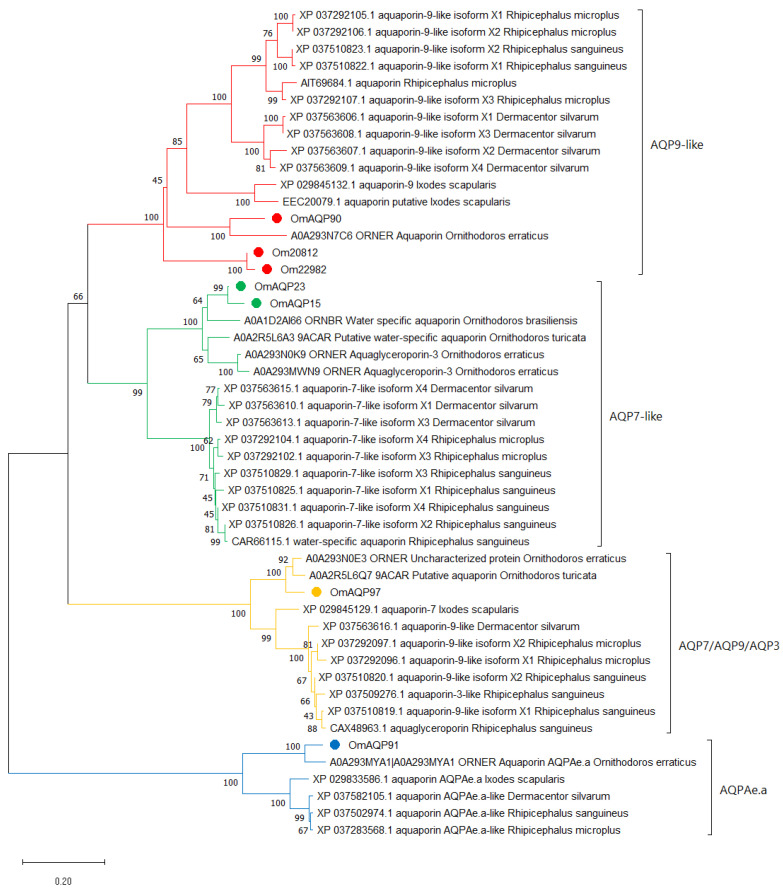
Neighbour-joining analysis of the phylogenetic relationship of *O. moubata* AQPs and their orthologues in other ticks. GeneBank or Uniprot accession numbers followed by the protein description and species name are shown, except for *O. moubata* AQPs. OmAQPs are identified by their simplified name and marked by red, green, yellow or blue dots depending on the clade in which they were grouped. Evolutionary distances were computed using the Poisson correction method. Branch support values (10,000 bootstraps) for the nodes are indicated.

Phylogenetic analysis of the seven OmAQPs and their top 42 orthologues grouped them with high confidence into four well-defined clades, which were referred to as AQP9-like, AQP7-like, AQP7/AQP9/AQP3 and AQPAe.a (Figure 2).

Clade AQP9-like included the OmAQP90, Om20812 and Om22982 isoforms, the XP_029845132.1 aquaporin-9 isoform X1 from *I. scapularis*, which appeared as a functional annotation associated with these three OmAQP transcripts and a range of sequences annotated as “AQP9-like”. This clade can be further divided into up to four subclades. Two of them comprise the 10 and 2 orthologous sequences found in metastriata and prostriata ticks, respectively, while the other two comprise the argasid sequences. One clade combines OmAQP90 with its homologue in *O. erraticus*, while the other brings together the Om20812 and Om22982 isoforms. Multiple sequence alignment of the AQPs in this clade showed highly conserved regions throughout the entire protein sequence, including the two NPA motifs, the amino acids that form the ar/R constriction, specifically tryptophan 51, isoleucine 180, methionine 188 and arginine 195, along with aspartic acid 196, immediately downstream of the second NPA motif (Figure S2). Altogether, these amino acids shape the aqueous pore and determine its permeability [25]. The aspartate residue downstream of the second NPA box enlarges and makes the pore more hydrophobic, allowing the transport of small molecules larger than water, such as glycerol. This aspartate residue is considered the signature key for aquaglyceroporins (gAQPs) [28]. Thus, OmAQP90, Om20812 and Om22982 can be classified as gAQPs, which is in accordance with the fact that human AQP9 also belongs to the gAQP subfamily and is known to transport a range of neutral solutes, including glycerol, urea and arsenite [25].

Clade AQP7-like included the OmAQP23 and OmAQP15 isoforms and the CAR66115.1 water-specific aquaporin from *Rhipicephalus sanguineus*, which appeared as a functional annotation associated with both isoforms and numerous sequences annotated as “AQP7-like”. This clade is clearly divided into two subclades, one comprising argasid sequences and the other comprising the ixodid orthologues (Figure 2). Multiple sequence alignment of AQPs in this clade showed very highly conserved regions throughout the entire protein sequence, including the two NPA motifs: the ar/R constriction, which in this clade was formed by tryptophan 54, alanine/isoleucine 193, glycine 201 and arginine 208; and the aspartic acid 209 downstream, the second NPA motif (Figure S3). As for the former clade, this aspartic acid residue indicates that this AQP7-like clade comprises members of the gAQP subfamily. In humans, AQP7 is classified as a member of the gAQP subfamily, and it is permeable to water, urea and arsenite [25].

Clade AQP7/AQP9/AQP3 included OmAQP97 and the CAX48963.1 aquaglyceroporin from *Rhipicephalus sanguineus*, which appeared as a functional annotation associated with OmAQP97, as well as several sequences annotated as AQP7, AQP9-like or AQP3-like. This clade is also clearly divided into two subclades, comprising argasid and ixodid sequences, respectively (Figure 2). Multiple sequence alignment of AQPs in this clade showed extensive conserved regions throughout the entire protein sequence, covering the two NPA motifs and the ar/R constriction, which in this clade was formed by tryptophan 58, cysteine 201, methionine 209 and arginine 216, as well as the aspartic acid 217 downstream of the second NPA motif (Figure S4). The presence of the aspartic acid residue in the second NPA box indicates that members of this clade are also aquaglyceroporins, as are human AQP9, AQP7 and human AQP3, with the latter known to transport glycerol and water [25].

Finally, clade AQPAe.a included OmAQP91 and the XP_029833586.1 aquaporin AQPAe.a-like from *Ixodes scapularis*, which appeared as a functional annotation associated with OmAQP91, as well as some additional sequences annotated as AQPAe.a-like. As for the two former clades, this one is further divided into two subclades, comprising argasid and ixodid sequences (Figure 2). Proteins in this clade also showed extensive conserved regions throughout the entire protein sequence, including the two NPA motifs and the ar/R constriction, comprised of phenylalanine 62, histidine 190, glycine 198 and arginine 205. In contrast, this clade lacks an aspartic acid residue downstream of the second NPA motif (Figure S5). The lack of this aspartate, together with the well-conserved amino-terminal NPA motif, indicates that the AQPs in this clade are members of the water-selective AQP subfamily [28]. This is in accordance with the function described for AQPAe.a, a member of the DRIP family of insect AQPs, which is considered homologous to the water-selective human AQP4 [25].

### 2.4. OmAQP Structure and 3D Modelling

**Figure 3:**
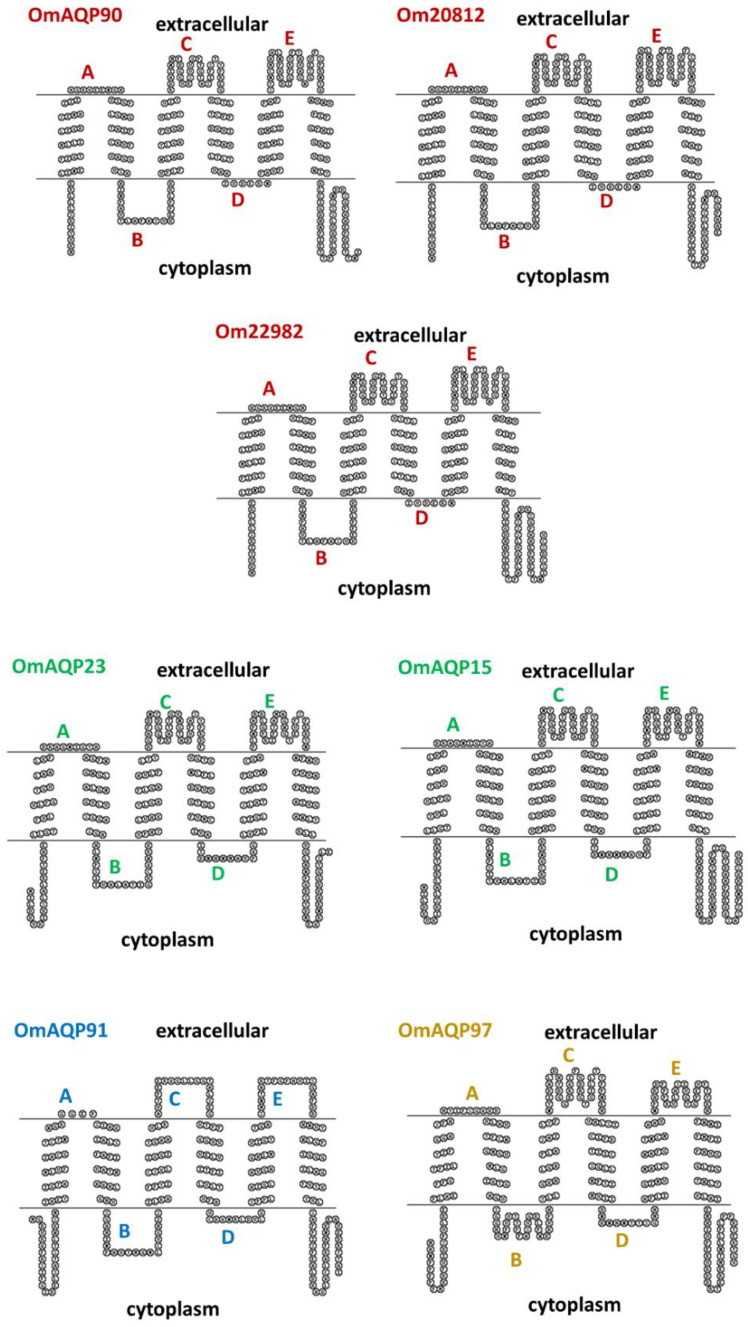
Topology predictions by SACS TMHMM server for the OmAQP monomers. All of them were predicted to have six transmembrane domains connected by five loops (indicated by letters). AQPs with the same colour belong to the same clade.

Topology predictions for the seven OmAQP proteins showed a highly conserved structure with six transmembrane domains and five connecting loops: three extracellular (A, C and E) and two cytoplasmic (B and D) (Figure 3). Among the extracellular loops, loop A is the shortest, with 4–10 amino acids, while loops C and E are longer, with 19–36 amino acids. Cytoplasmic loops B and D had lengths from 20 to 26 and 6 to 12 amino acids, respectively.

**Figure 4:**
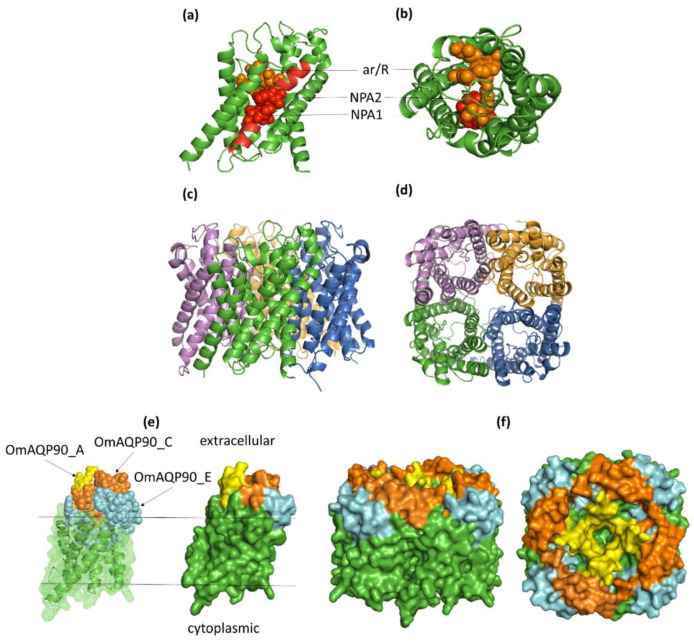
Three-dimensional models for OmAQP90. (**a**,**b**) Side and top view, respectively, of the monomer cartoon showing the position of the two NPA motifs (red spheres) and the aromatic arginine (ar/R) constriction (orange spheres); (**c**,**d**) side and top view of the tetramer cartoon model; (**e**) monomer mesh and surface model showing the exposure of the 3 antigenic peptides on the extracellular surface of the molecule: OmAQP90_A yellow spheres, OmAQP90_C orange spheres and OmAQP90_E cyan spheres; (**f**) side and top view of tetramer surface model showing peptide exposure.

Although each AQP monomer functions as a single channel pore, AQPs form tetramers in biological membranes [26]; accordingly, monomeric and tetrameric protein 3D models were constructed for all of the *O. moubata* AQPs analysed. The templates used for homology modelling, as well as the evaluation of the quality of the OmAQP 3D models are shown in Table S3. OmAQP models showed highly conserved 3D structures, as can be observed in Figure 4 and Figures S6–S11. Figure 4 shows the 3D models generated for OmAQP90, which can be taken as representative of all of the OmAQPs analysed. The OmAQP90 3D monomer shows the characteristic tertiary structure of AQPs resembling an hourglass, which is formed by the six transmembrane alpha-helices and loops B and E, which form additional short hydrophobic helices that dip halfway into the membrane from opposite sides. In this way, the two NPA boxes, located at loops B and E, face each other at the centre of the hourglass, shaping the AQP pore and its substrate selectivity. The ar/R constriction is located above the NPA filter, towards the extracellular side of the pore mouth, which also contributes to the pore shape and substrate selectivity (Figure 4a,b). Tetrameric 3D models representing the AQP assembly in biological membranes are shown in Figure 4c,d.

### 2.5. Predicted B and T Cell Epitopes and Epitope Exposure; Antigenic Peptide Candidates

For each of the seven OmAQPs analysed, detailed information on their predicted physicochemical and structural features, including putative epitope exposure, as well as predicted B and T cell epitopes, can be accessed in Tabels S4–S10.

As can be observed in OmAQP90 (Table S4), the three predicted extracellular domains (loops A, C and E) showed low structural complexity and predicted surface exposure, as well as high ratios of amino acid residues with scores above the thresholds for beta turns, flexibility and hydrophilicity, which indicates a high probability of being part of an epitope. 

The B cell epitope predictors provided different sets of overlapping linear B cell epitopes that cover these extracellular domains, as well as several non-linear B-cell epitopes also located on the extracellular domains (loops A, C and E), indicating that they could be presented directly to B cells and induce a humoral response. In addition, these fragments show predicted proteasome cut sites that could generate peptides that can bind to MHC I and II molecules and be potentially presented to T cells. These predictions make the three extracellular sequence fragments of OmAQP90 promising candidates for the synthetic production and testing of protective efficacy in future vaccine trials (Table 3).

Similar results were obtained after analysis of the six additional OmAQPs (Tables S5–S10), providing each of them with a set of three potential antigenic peptides and a whole set of 21 peptide candidates (Table 3, Figures S2–S5).

The antigenic peptides were arranged in Table 3 according to the extracellular loop they were designed from and then grouped in clades, which allows the similarity of their sequences to be compared. For each loop, it can be seen that the peptide sequences were different between clades but highly conserved or identical inside each clade. This leaves 15 unique antigenic peptides, five per loop: two resulting from clade AQP9-like and one resulting from each of the other three clades. In other words, there are four sets of unique peptides: the set from clade AQP9-like includes six different peptides, and the sets from the other clades include three peptides each (Table 3).

Interestingly, the peptides derived from loops C and E are highly conserved in orthologous AQPs from the tick species inside each clade, in particular in clades AQP7-like, AQP7/AQP9/AQP3 and AQPAe.e (Figures S2–S5), suggesting epitope conservation among tick species.

Mapping the antigenic peptides to the 3D model of their corresponding OmAQP show that the peptides derived from loops A and C are entirely exposed on the extracellular surface of the proteins, while the peptides derived from loop E are partially exposed on the extracellular protein surface and partially buried. This is because the N-terminal half of these peptides, which contains the carboxyterminal NPA motif (Table 3), partially dip into the membrane to form the NPA filter (Figure 4e,f; Figures S6–S11, panels e and f).

### 2.6. Physicochemical, Allergenic and Toxic Properties of Selected Antigenic Peptides

The ten antigenic peptides derived from loops C and E were selected due to their larger size and higher conservation inside clades; they were further analysed to predict their physicochemical properties and harmful properties to the host. Detailed information on these predictions is shown in Table S11.

All of the peptides exhibited mostly desirable characteristics regarding stability, thermal stability, hydrophilicity and solubility, as well as moderately acidic to neutral pIs. In addition, they all were predicted as non-allergens and as non-haemolytic, toxic or anti-angiogenic molecules, hence having no negative impacts on the host.

For comparison, Table S11 also includes the same predictions for the antigenic peptides derived from the extracellular domains of *R. microplus* AQP2 (RmAQP2), which were successfully utilised to immunise cattle by Scoles et al. [35]. These peptides are in the same range of stability, thermal stability, solubility and pI as those designed from *O. moubata*, although they are more hydrophobic. They present no harmful properties to the host either, except one of them that can be anti-angiogenic.

## 3. Discussion

As stated in the introduction, AQPs are considered promising targets for anti-tick vaccine development due to their important biological functions in these parasites [30], their immunogenicity [31,34,35,38] and their accessibility to host antibodies in the midgut and in other organs bathed with haemolymph, such as salivary and coxal glands.

Accordingly, we aimed to identify the AQP family members expressed by female *O. moubata* ticks and to assess their potential as vaccine targets. For this, we took advantage of the recent availability of the *O. moubata* midgut and salivary gland transcriptomes obtained from female ticks taken before and after feeding [18,21]. The scrutiny of these transcriptomic datasets allowed the identification, cloning and characterisation of up to seven full-length different AQP transcripts/proteins. Given that our analysis was not genome-wide, the possibility that *O. moubata* expresses additional AQP genes in other organs or tissues that were not included in this study, such as the Malpighian tubules, ovaries or synganglion, cannot be ruled out. Despite this limitation, the number of AQP genes identified herein in *O. moubata* was inside the range of six to eleven AQP genes identified in most of the insects and ticks studied for AQPs to date [28,30]; furthermore, it represents the first comprehensive analysis of the AQP family members expressed in argasid ticks.

After the identification of these seven OmAQPs, we studied their tissue expression pattern in the tick organs directly involved in the processes of tick feeding, blood concentration and excess water excretion, which are basically the salivary glands, midgut and coxal glands in argasid ticks [30,39]. In contrast with the previous results of RNA-seq, which identified five OmAQPs in the midgut, four in the salivary glands and two in both organs (Table 1), the results in Figure 1 showed that all of the identified OmAQPs are expressed in the midgut and salivary glands, but only three are expressed in the coxal glands. This result suggests that all of these OmAQPs may be involved in the production of saliva by the salivary glands and in the concentration of the blood meal in the midgut, but only three of them (OmAQP90, OmAQP97 and OmAQP23) would be involved in the excretion of the resulting excess water via coxal glands. Given the small volume of salivary fluid (5–10 µL) produced by *O. moubata* females during feeding [20] compared to the large volume of coxal fluid that is excreted during and immediately after feeding (close to 500 µL, personal observation), the higher number of OmAQPs theoretically involved in saliva production compared to those potentially involved in coxal fluid excretion is noteworthy. This suggests that the expression levels, activity and functions of OmAQPs would most likely be differentially regulated in these organs throughout the tick trophogonic cycle. According to previous data [18,21], most of these OmAQPs are either constitutively expressed or upregulated in the midgut and salivary glands upon feeding. These data are unknown for coxal glands, but it can be hypothesised that OmAQP expression in coxal glands would be transiently upregulated during and immediately after blood feeding in response to the increased volume of water in haemolymph. As the tick specimens used herein for RNA extraction were unfed, it could be assumed that OmAQP expression in the coxal glands was not upregulated, with only the constitutively expressed OmAQP genes being detected. Obviously, demonstration of this hypothesis would require further experimental studies. Additionally, OmAQPs might be involved in more numerous and varied functions in salivary glands than in coxal glands, which would require the expression of more numerous sets of OmAQPs. In fact, the salivary glands produce not only the complex cocktail of pharmacologically active molecules that facilitate blood feeding [40] but also ion-enriched saliva that is secreted on the gnathosoma during the off-host periods, which allows ticks to absorb environmental water vapour and subsequently ingest this water-enriched fluid avoiding dehydration [41]. The potential role of AQPs in this process is unknown, but their expression in the salivary glands suggests they are most likely involved in the osmoregulation of tick saliva and water vapour uptake [30].

Former AQP-based anti-tick vaccine trials targeted only one or two AQP proteins, obtaining partial protection [31,34,35,38]. As ticks express multiple AQPs, the vaccine-induced loss of function of the targeted AQP may have been compensated by other AQPs playing overlapping or redundant functions. This suggests that it would be necessary to neutralise the function of as many OmAQPs as possible, ideally of all of them, to obtain a fully protective AQP-based anti-tick vaccine. Previous works also showed that a full-length expressed AQP is not required to induce a protective immune response since targeting the extracellular peptide domains is sufficient to induce an immune response able to interfere with AQP function and reduce overall tick fitness and survival [34,35].

Based on this notion, we undertook the characterisation of the seven AQPs detected in *O. moubata* to identify the AQP peptide domains that can be proposed as potential vaccine targets. To this end, we first aligned the OmAQPs and analysed their phylogenetic relationships. This analysis allowed the functional classification of OmAQPs and revealed that they are grouped into four distinct and well-supported clades (Figure 2), which showed notable differences in sequence among clades, but high sequence conservation inside clades (Table S2, Figures S2–S5). This result evidenced the absence of sequence motifs conserved in all OmAQPs, which otherwise could have been proposed as vaccine targets to simultaneously neutralise the entire set of OmAQPs, but suggests the possibility that there may be clade-specific conserved sequence motifs that would allow the simultaneous neutralisation of all members in the clade.

Three of these clades, AQP9-like, AQP7-like and AQP7/AQP9/AQP3, were classified as aquaglyceroporins (gAQPs) and included six of the seven OmAQPs found. Only clade AQPAe.a and its unique member (OmAQP91) were classified as a water-selective aquaporin (wAQP), while no super-aquaporins (sAQP) were found in *O. moubata*. Since no functional assays were performed in this study, this functional classification and ensuing discussion must be taken with caution. The classification was based only on the primary structure of OmAQPs and followed the algorithm of Ishibashi et al. [28]. This algorithm considers that the presence of the aspartic acid residue immediately downstream of the second NPA motif is the signature sequence for gAQPs, while the absence of this aspartate together with conservation of the first NPA motif is the signature sequence for wAQPs. Finally, the absence of aspartate together with a poorly conserved first NPA motif and a cysteine residue at the nine residues downstream of the second NPA motif would be the signature sequence for sAQPs. This highly biased OmAQP distribution towards gAQPs is remarkable since early arthropods and ticks tend to express more balanced gAQP/wAQP ratios and to express at least one member of the sAQP subfamily as well, whereas the more advanced insects (hexapoda) have lost their gAQPs [28]. It could be speculated that the reduction in wAQPs in *O. moubata* might be related to the prevention of water loss during the off-host periods between blood-feeding events. Conversely, the expansion of gAQPs might allow the accumulation of glycerol and osmolytes in cells during this period, protecting cells from dehydration, as has been suggested for other blood-feeding arthropods [30]. This would facilitate *O. moubata* tick survival in environments with low relative humidity in their wide distribution area [8]. Additionally, *O. moubata* gAQPs might also be involved in the uptake of glycerol as a nutrient for carbohydrate metabolism. This has been observed for some bacterial gAQPs [42], and it was described as an additional function for human AQP9, which is involved in the uptake of glycerol from plasma into the hepatocytes for gluconeogenesis [43].

Regardless of the potential functions of OmAQPs, their structural analysis and 3D modelling confirmed that loops A, C and E were the only extracellular domains potentially exposed to host antibodies ingested with blood and, therefore, those of interest as vaccine targets. In parallel, the epitope predicting tools revealed that these loops concentrate overlapping B and T cell epitopes able to induce humoral and cellular immune responses, confirming the potential of these peptide domains as vaccine targets.

Accordingly, a set of three potential antigenic peptides was designed from loops A, C and E of each OmAQP and their sequences compared inside each clade, which allowed the detection of redundant peptides and the reduction in the initial set of 21 antigenic peptides to 15 unique peptides (Table 3). Additionally, the peptides designed from loops A were removed as vaccine targets at this stage because they were less conserved inside each clade than the peptides designed from loops C and E and because of their shorter length (9–11 amino acids), which anticipates potentially lower immunogenicity.

This last selection step kept the antigenic peptides derived from loops C and E as candidates, which were then examined to predict their physicochemical characteristics and damaging properties to the host. This examination showed that they were physicochemically stable, non-allergenic and non-harmful to the host. Hence, ten peptides that showed most of the desired characteristics were identified, which can be tested in multicomponent vaccines aimed to target and neutralise the entire set of AQPs expressed by *O. moubata*. In addition, given that these peptides showed high sequence conservation to other tick species inside the same clade (Figures S2–S5), vaccination with these peptides could result in some degree of cross-reactivity and protection against other ticks, as was previously observed among *O. erraticus* and *O. moubata* [34].

Animal vaccination trials are thus the necessary next step to validate the immunogenicity and protective efficacy of these peptides and to explore the influence of different aspects such as the peptide production method (either conjugated to protein carriers or as synthetic oligomers to enhance their immunogenicity) [44], their formulation (combined, alone) and administration (route, dose, adjuvant). Using peptide domains as vaccine targets may make it easier to include multiple targets to design multivalent vaccines, and it will reduce the cost of a putative vaccine since the smaller the biomolecule, the easier it is to synthesise and store.

## 4. Materials and Methods

### 4.1. Selection of Transcripts Containing AQP Coding Sequences

The *O. moubata* midgut and salivary glands transcriptomes obtained in previous works, which are available under Bioprojects number PRJNA377416 (TSA: GFJQ00000000) and PRJNA667315 (TSA: GIXP00000000) [18,21], were scrutinised for transcripts annotated as aquaporins. The AQP transcripts found were manually inspected, and only those containing predicted full-length open reading frames (ORF) for aquaporins, i.e., around 800–900 nucleotides and 270–300 amino acids, were selected (Table S1). After that, transcripts with identical sequences were filtered, and only unique sequences were used in subsequent analyses (Table 1).

### 4.2. Ticks and Tick Material

The *O. moubata* ticks used in this study were obtained from the pathogen-free laboratory colony that has been maintained in the IRNASA-CSIC (Salamanca, Spain) since the 1990s at 28 °C, 85% relative humidity, a 12 h light/dark photoperiod and regularly fed on New Zealand White rabbits [21]. 

Fifteen unfed *O. moubata* female ticks were dissected in the cold (4 °C) phosphate-buffered saline (PBS), pH 7.4, treated with 0.1% diethyl pyrocarbonate (DEPC). Tick midguts, salivary glands and coxal glands were extracted separately and immediately pooled in RNAlater solution (Sigma, St. Louis, MO, USA) in three pools, each containing a different type of organ.

Total RNA was purified from the RNA later-stabilised samples using the PureLink™ RNA Mini Kit (Invitrogen, Carlsbad, CA, USA) following the manufacturer’s instructions and preserved at −80 °C.

Complementary DNA (cDNA) was synthesised from total RNA samples using the First Strand cDNA Synthesis Kit for RT-PCR (AMV) (Roche, Basel, Switzerland) and the oligo (dT)15 primer according to the manufacturer’s instruction and stored at −80 °C until its use for PCR amplification.

### 4.3. AQP Sequence Verification and Tissue Expression: PCR Amplification, Cloning and Sequencing

The coding sequences of *O. moubata* AQPs (OmAQPs) were amplified by PCR using specific primer pairs designed ad hoc from the corresponding transcript sequences (Table S1). The primers were designed using the Primer3Plus software (https://www.bioinformatics.nl/cgi-bin/primer3plus/primer3plus.cgi, accessed on: 6 September 2021) [45]. Table 4 shows the specific primer pairs and the PCR conditions for these amplifications.

PCR amplifications of OmAQPs were performed from cDNA samples of the midgut, salivary glands and coxal glands. The PCR products were electrophoresed in 1% agarose gels stained with GelRed (Biotium, Fremont, CA, USA), purified from gels using the StrataPrep DNA Gel Extraction Kit (Agilent Technologies, Santa Clara, CA, USA) and their concentration estimated by spectrophotometry at 260 nm (NanoDrop 2000, ThermoScientific, Waltham, MA, USA). The AQP cDNAs were cloned into the pSC-A sequencing vector using the StrataClone PCR Cloning kit (Stratagene, Santa Clara, CA, USA) according to the manufacturer’s instructions. The recombinant plasmids were transformed into *Escherichia coli* SoloPack cells, and the cells were plated on agar plates containing 100 µg/mL ampicillin, 25 μg/mL kanamycin and 80 µL of 2% X-gal and incubated overnight at 37 °C. Several recombinant colonies from each transformation were grown in 5 mL cultures of Luria–Bertani (LB) medium with 100 µg/mL ampicillin and 25 μg/mL kanamycin overnight at 37 °C. The cells were harvested, lysed and plasmid DNA purified using the Qiaprep Spin Miniprep kit (Qiagen, Hilden, Germany). Plasmids were digested with EcoRI (Promega Biotech Ibérica, Alcobendas, Madrid, Spain) for 2 h at 37 °C and electrophoresed in 1% agarose gel to verify the presence of the expected insert. After that, inserts were sequenced in both strands using the primers T3 and T7 at the DNA Sequencing service of the Nucleus platform, University of Salamanca (Spain).

At least three clones of each recombinant AQP from each tissue sample were sequenced to verify the correctness and integrity of the sequences. The resulting nucleotide sequences were handled and compared to the corresponding transcript sequence using the Chromas 2.6.2 and Multalin tools (http://multalin.toulouse.inra.fr/multalin/, accessed on: 4 October 2021) [46].

### 4.4. Prediction and Analysis of the AQP Amino Acid Sequences

The amino acid sequences of OmAQPs were obtained using the ‘Translate’ tool available on the ExPASy server (http://web.expasy.org/translate/, accessed on: 5 October 2021) and analysed as follows. Their isoelectric point (pI) and molecular weight (MW) were calculated using https://web.expasy.org/compute_pi/ (accessed on: 5 October 2021). The presence of secretion signals and glycosylphosphatidylinositol (GPI) anchor sites was examined using SignalP 4.0 (http://www.cbs.dtu.dk/services/SignalP, accessed on: 5 October 2021) [47] and the PredGPI predictor (http://gpcr.biocomp.unibo.it/predgpi/pred.htm, accessed on: 5 October 2021), respectively [48]. The N- and O-glycosylation sites were predicted using the https://services.healthtech.dtu.dk/service.php?NetNGlyc-1.0 (accessed on: 5 October 2021) [49] and https://services.healthtech.dtu.dk/service.php?NetOGlyc-4.0 (accessed on: 5 October 2021) [50] servers. Additionally, antigenicity prediction was performed with the VaxiJen 2.0 tool using the 0.5 antigenicity threshold established by default for parasites (http://www.ddg-pharmfac.net/vaxijen/vaxijen/vaxijen.html, accessed on: 5 October 2021) [51].

### 4.5. Phylogenetic Analysis

Orthologous sequences of each OmAQP in the Argasidae and Ixodidae families were searched by BLASTp in the Uniprot and NCBInr databases. Orthologues showing an E value < 10–110, more than 85% sequence coverage and 50–100% sequence identity were selected. Redundant orthologues, i.e., those that matched more than one OmAQP, were removed.

Phylogenetic and molecular evolutionary analyses of OmAQPs and their tick orthologues were conducted with MEGA version 11 [52]. Sequence alignment was performed by Muscle using default parameters, and phylogenetic analysis was conducted using the neighbour-joining method. Gaps were treated as pairwise deletions, amino acid distances were calculated using a Poisson model, and branch supports were estimated using bootstrap analysis (10,000 bootstraps).

### 4.6. Protein Structure and Epitope Exposure

Topographical analyses of OmAQPs were performed using the TMHMM 2.0 (https://services.healthtech.dtu.dk/service.php?TMHMM-2.0, accessed on: 6 October 2021) and SACS TMHMM (http://www.sacs.ucsf.edu/cgi-bin/tmhmm.py, accessed on: 6 October 2021) [53,54] servers in order to define their transmembrane domains and extracellular exposed regions.

A number of physicochemical and structural features of the protein sequences that are known to correlate with the amino acids’ probability of being part of an epitope were predicted for each OmAQP using the IEDB Analysis Resource tools (http://tools.iedb.org/bcell/, accessed on: 7 October 2021) with the settings and thresholds established by default. The surface accessibility or probability of an amino acid found on the protein surface was assessed using the Emini prediction method [55] and the surface frame of BepiPred 2.0 [56]. The presence of beta turns within the amino acid sequence, the flexibility of the protein segments and the amino acid hydrophilicity is related to a high probability of amino acids being part of an epitope. These features were predicted using the Chou and Fasman beta-turn prediction method [57], the Karplus–Schultz scale of flexibility [58] and the Parker scale of hydrophilicity [59].

Secondary structure prediction and 3D modelling of OmAQPs were performed using the Phyre2 (http://www.sbg.bio.ic.ac.uk/phyre2, accessed on: 18 October 2021) [60] and Swiss model (https://swissmodel.expasy.org/, accessed on: 19 October 2021) servers. Model quality was checked with ProSA (https://prosa.services.came.sbg.ac.at/prosa.php, accessed on: 6 June 2022) [61] and PROCHECK [62], which was accessed through Saves v6.0 (https://saves.mbi.ucla.edu, accessed on: 6 June 2022). The 3D models were visualised and handled with the Pymol package [63].

### 4.7. Prediction of B and T Cell Epitopes

The presence of linear B-cell epitopes in OmAQPs was predicted using the following tools: (i) the Kolaskar and Tongaonkar method [64]; (ii) the BepiPred 1.0 [65] and (iii) the BepiPred 2.0 (epitope frame) programs, which were accessed through the IEDB Analysis Resource tools (http://tools.iedb.org/bcell/, accessed on: 8 November 2021); (iv) the ABCpred server (http://www.imtech.res.in/raghava/abcpred/index.html, accessed on: 8 November 2021) [66] and (v) the BCEpred server (http://www.imtech.res.in/raghava/bcepred/, accessed on: 8 November 2021) [67]. The presence of discontinuous B-cell epitopes in OmAQPs was predicted using the Ellipro tool [68], which was accessed through the IEDB Analysis Resource tools (http://tools.iedb.org/bcell/, accessed on: 7 June 2022).

The presence of T-cell epitopes in OmAQPs was predicted using the TepiTool program accessed through the IEDB Analysis Resource tools (http://tools.iedb.org/tepitool/, accessed on: 9 November 2021). Information on MHC alleles from pigs and other vertebrate hosts of *O. moubata* is currently limited to pig MHC class I; accordingly, the available, well-known human and mouse MHC class II allelic datasets were used to extrapolate the vertebrate host with unknown MHC-II alleles such as pig. Hence, T-cell epitopes with affinity to pig MHC class I molecules were predicted by applying the settings established by default. T-cell epitopes with an affinity for human MHC-II were predicted using the preselected 7-allele method and the default settings established for this method. Finally, T-cell epitopes with an affinity for mouse MHC-II were predicted using the H2-IAb, H2-IAb and H2-IEd preselected alleles, also applying default settings.

Additionally, the prediction of the OmAQPs proteasomal processing was obtained to check if the epitopes generated can be MHC binders and thus presented to the host immune system. For this, the NetChop 3.1 (https://services.healthtech.dtu.dk/service.php?NetChop-3.1, accessed on: 9 November 2021) [69] and IPCPS (http://imed.med.ucm.es/Tools/pcps/, accessed on: 9 November 2021) programs were used.

### 4.8. Prediction and Analysis of Antigenic Peptides

Potential antigenic peptides were predicted from the OmAQP domains meeting the following characteristics: (i) extracellular and exposed on the protein surface, (ii) low structural complexity and (iii) overlapping B and T cell epitopes.

The sequence regions meeting these criteria were proposed as potential antigenic peptides for vaccines, and their physicochemical characteristics and potentially harmful effects on the host were predicted as follows.

Their molecular weight (Da), theoretical isoelectric point (pI), instability index, aliphatic index and grand average of hydropathicity (GRAVY) were assessed using the Expasy ProtParam server (http://expasy.org/cgi-bin/protpraram, accessed on: 7 March 2022). Peptide solubility and crystallisation propensity were predicted using Protein-Sol (https://protein-sol.manchester.ac.uk/, accessed on: 7 March 2022) [70] and CRYSTALP2 (http://biomine.cs.vcu.edu/servers/CRYSTALP2/, accessed on: 7 March 2022), respectively [71]. The allergenicity of the peptides was predicted using the online servers AllergenFP (https://ddg-pharmfac.net/AllergenFP/, accessed on: 7 March 2022) [72], AllerTop (https://www.ddg-pharmfac.net/AllerTOP/, accessed on: 7 March 2022) [73] and AllerCatPro (https://allercatpro.bii.a-star.edu.sg/, accessed on: 7 March 2022) [74]. The haemolytic and toxic properties of the selected peptides were analysed with HemoPI (https://webs.iiitd.edu.in/raghava/hemopi/, accessed on: 7 March 2022) [75] and ToxinPred (https://webs.iiitd.edu.in/raghava/toxinpred/index.html, accessed on: 7 March 2022) [76] using all SVM prediction methods in both servers. Finally, the anti-angiogenic capacity of peptides was predicted with AntiAngioPred using the NT15 AAC and whole peptide AAC prediction methods (https://webs.iiitd.edu.in/raghava/antiangiopred/index.html, accessed on: 7 March 2022) [77].

For comparison, all of these analyses were also performed for the antigenic peptides derived from the extracellular domains of *R. microplus* AQP2 (RmAQP2), which were successfully utilised by Scoles et al. [35] to immunise cattle.

## 5. Conclusions

Tick AQPs are promising targets for tick vaccines due to their involvement in vital physiological processes of ticks, their immunogenicity and the ease of access by host antibodies ingested with blood, which can therefore neutralise the aquaporin function and impact tick reproduction and survival. This is the first study aimed at the systematic identification and characterisation of the AQPs expressed in an argasid tick, *O. moubata*, which is the main African vector of ASF and TBRF, as vaccine targets. The scrutiny of available *O. moubata* transcriptomic datasets followed by PCR amplification and cloning identified seven unique AQP coding sequences, which reflect a number of genes similar to the usual range of six to eleven AQP genes identified in most insects and ticks. The functional classification of OmAQPs indicates that six of them are aquaglyceroporins, and only one is a water-selective aquaporin. Tissue expression analysis confirmed that all the seven OmAQPs are expressed in the tick salivary glands and midgut, but only three are expressed in the coxal glands, suggesting more complex and varied functions for OmAQPs in the physiological processes that take place in salivary glands and the midgut. These seven OmAQPs are grouped into four well-defined clades that show low sequence similarity among clades but high sequence conservation inside each clade. Protein topology and structure analysis, 3D modelling and epitope prediction for each OmAQP showed that the three extracellular peptide domains (loops A, C and E) are accessible to host antibodies and concentrate overlapping B and T cell epitopes, which are capable of inducing humoral responses, making them interesting vaccine targets. Accordingly, based on the amino acid sequences of these extracellular domains, a set of ten antigenic peptides was designed and characterised in silico, which showed the adequate properties to be produced and tested in animal vaccine trials aimed at evaluating their immunogenicity and protective efficacy against tick infestations. Their administration as a multicomponent vaccine is expected to neutralise the whole set of AQPs expressed by *O. moubata* and to provide high anti-tick protective efficacy. Should this be demonstrated, it will contribute to increasing the scarce number of protective antigens identified hitherto in argasid ticks.

## Figures and Tables

**Table 1:** Unique transcripts containing full-length ORFs for aquaporin proteins recovered from the transcript datasets of *O. moubata* midgut (M) and/or salivary glands (SG). The transcript identification code, protein accession code (GenBank/Uniprot) and their annotation in the *O. moubata* transcriptomes are indicated. A simplified name and the ORF lengths in base pairs (bp) are also included.

List	Transcript Dataset	Transcript Code	Protein Accession (GenBank/Uniprot)	Name	ORF Length (bp)	Match_Sequence	Species	Protein Name
1	M	ci|000144090	-	OmAQP90	867	XP_029845132.1	*I. scapularis*	aquaporin-9 isoform X1
2	SG	OM_20812	MBZ3958194	Om20812	843			
3	SG	OM_22982	MBZ3960076	Om22982	882			
4	M, SG	ci|000124891, OM_7339	A0A1Z5L1C7	OmAQP91	816	XP_029833586.1	*I. scapularis*	aquaporin AQPAe.a-like
5	M	ci|000113997	A0A1Z5L6U6	OmAQP97	879	CAX48963.1	*R. sanguineus*	aquaglyceroporin
6	M, SG	ci|000114723, OM_21119	A0A1Z5L547	OmAQP23	849	CAR66115.1	*R. sanguineus*	water-specific aquaporin
7	M	ci|000148315	A0A1Z5KVQ3	OmAQP15	915			

**Table 2:** Molecular characterisation of *O. moubata* AQP proteins identified in this study. The AQPs amino acid sequences were inspected for molecular weight (MW), isoelectric point (pI), signal peptide, glycosylphosphatidylinositol (GPI) anchor sites, N- and O-glycosylation sites and global antigenicity (VaxiJen score).

List	Name	Tissue Expression	GenBank/Uniprot Code	Protein Length (aa)	MW (kDa)	pI	Signal P	GPI Anchor	N-glycosilation Sites	O-glycosilation Sites	VaxiJen Score
1	OmAQP90	SG, M, CG	-	288	31.2	6.4	no	no	no	128T, 132S	0.5979
2	Om20812	SG, M	MBZ3958194	280	30.1	5.4	no	no	no	126T, 128T	0.6173
3	Om22982	SG, M	MBZ3960076	293	31.4	5.1	no	no	no	126T, 128T	0.6597
4	OmAQP91	SG, M	A0A1Z5L1C7	271	28.4	6.0	no	no	no	no	0.3729
5	OmAQP97	SG, M, CG	A0A1Z5L6U6	292	32.6	7.2	no	no	no	no	0.5386
6	OmAQP23	SG, M, CG	A0A1Z5L547	282	30.3	7.7	no	no	no	no	0.5234
7	OmAQP15	SG, M	A0A1Z5KVQ3	304	32.7	7.6	no	no	no	no	0.5007

**Table 3:** Antigenic peptides derived from the *O. moubata* AQPs. One peptide per extracellular domain of each OmAQP was designed. For each loop, the peptides designed from OmAQPs in the same clade showed highly conserved (purple) or identical (red) sequence. The NPA motif in peptides from loop E is highlighted in bold. B, peptide-containing B-cell epitopes. T, peptide-containing T-cell epitopes.

List	Extracellular Domain	Clade	AQPs in the Clade	Peptide Name	Peptide Sequence	Peptide Length	Peptide Position	Peptide Type
1	Loop A	AQP9-like	OmAQP90 Om20812 Om22982	OmAQP90_A Om20812_A Om22982_A	AGRQEENGH AGRQEHNAG AGRQEHNAG	9 9 9	36–44 34–42 34–42	B B B
2								
3								
4		AQP7-like	OmAQP23 OmAQP15	OmAQP23_A OmAQP15_A	KFDRAGNIGYA KFDRAGNIGYA	11 11	38–48 38–48	B, T B, T
5								
6		AQP7/AQP9/AQP3	OmAQP97	OmAQP97_A	HYIFSGQKD	9	42–50	B
7		AQPAe.a	OmAQP91	OmAQP91_A	CGTCTNWGRGGEPSIA	16	42–56	B, T
8	Loop C	AQP9-like	OmAQP90 Om20812 Om22982	OmAQP90_C Om20812_C Om22982_C	RDAINMFDGGVRSVVGPTGTASIFSTYPREG RDAIDAVDSGVRSVLGPTGTAPIFATYPREG RDAIDAVDSGVRSVLGPTGTAPIFATYPREG	31 31 31	111–141 109–139 109–139	B, T B, T B, T
9								
10								
11		AQP7-like	OmAQP23 OmAQP15	OmAQP23_C OmAQP15_C	KGAFDNYDGGFRATTGVNGTADVFASYPRDF KGAFDNYDGGFRATTGVNGTADVFASYPRDF	31 31	115–145 115–145	B, T B, T
12								
13		AQP7/AQP9/AQP3	OmAQP97	OmAQP97_C	NYIDALDHYDGGERQIFGDRGTGILLTTFPNEH	33	117–149	B, T
14		AQPAe.a	OmAQP91	OmAQP91_C	AVTPEERQGLLGGTALSEGVTPFQG	25	120–144	B, T
15	Loop E	AQP9-like	OmAQP90 Om20812 Om22982	OmAQP90_E Om20812_E Om22982_E	SYNCMAAL**NPA**RDIGPRVFTAVAGWGSEVFSFRNYQ SYNCMAPL**NPA**RDLGPRVFTAIAGWGMEVFSVRDY SYNCMAPL**NPA**RDLGPRVFTAIAGWGMEVFSVRDY	36 35 35	194–229 193–227 193–227	B, T B, T B, T
16								
17								
18		AQP7-like	OmAQP23 OmAQP15	OmAQP23_E OmAQP15_E	PL**NPA**RDLGPRIFTAMAGWGTEVFSFRDYN PL**NPA**RDLGPRIFTAMAGWGTEVFSFRDYN	30 30	204–233 204–233	B, T B, T
19								
20		AQP7/AQP9/AQP3	OmAQP97	OmAQP97_E	**NPA**RDFPPRVLASIVGYGPEVFTYRH	26	210–235	B, T
21		AQPAe.a	OmAQP91	OmAQP91_E	ASM**NTA**RTFGPAVISGAFDDH	21	195–215	B, T

**Table 4:** Primers and PCR conditions used for amplification of the cDNAs coding AQP proteins.

List	Transcript Code	Primer Name	Primer Sequence (5′-3′)	Product Size (bp)	Tm
1	ci|000144090	OmAQP90F OmAQP90R	ATGAAGGTGTACATTCGGAGTC CTAGATGTTGGTTGTCTCTTTGG	867	57 °C
2	OM_20812	Om20812_22982F Om20812R	ATGAAGATACAGAGCACATTCGTC TCACAGCCAAGTTGGGCCTA	843	60 °C
3	OM_22982	Om20812_22982F Om22982R	ATGAAGATACAGAGCACATTCGTC CTATACACAACTATCGCAGCTGAAT	882	60 °C
4	ci|000124891 (OM_7339)	OmAQP91F OmAQP91R	ATGGGCCGTGTTCGCCAATT TCAGATGGCCGTGGTGCG	816	62.8 °C
5	ci|000113997	OmAQP97F OmAQP97R	ATGGCAAACCCACCGTTCC TTAGACACCGGTCTTTTCTGTCC	879	61 °C
6	ci|000114723 (OM_21129)	OmAQP23_15F OmAQP23R	ATGATTCTGGATAAAGTGAAGATTAAG TCATTCGAGGGAATACCCAC	849	59 °C
7	ci|000148315	OmAQP23_15F OmAQP15R	ATGATTCTGGATAAAGTGAAGATTAAG CTAGACCTTCGACTGTTTTTCGT	915	59 °C

PCR conditions were as follows: 94 °C for 3 min; 35× (94 °C for 15 s + Tm for 30 s + 72 °C for 40 s); 72 °C for 7 min.

## Data Availability

The *Ornithodoros moubata* midgut and salivary glands transcriptomic data are available under Bioprojects number PRJNA377416 (TSA: GFJQ00000000) and PRJNA667315 (TSA: GIXP00000000).

## Supplementary Materials

The following supporting information can be downloaded at: https://www.mdpi.com/article/10.3390/pathogens11060694/s1. Figure S1: Nucleotide (a) and amino acid (b) sequences of the seven *Ornithodoros moubata* AQPs amplified by PCR. (c) Nucleotide sequence alignments of the amplified *O. moubata* AQPs with the corresponding transcripts as obtained by RNA-seq; Figure S2: Alignment of the amino acid sequences included in clade AQP9-like. Figure S3. Alignment of the amino acid sequences included in clade AQP7-like; Figure S4: Alignment of the amino acid sequences included in clade AQP7/AQP9/AQP3; Figure S5: Alignment of the amino acid sequences included in clade AQPAe.a; Figure S6: 3D models for Om20812; Figure S7: 3D models for Om22982; Figure S8: 3D models for OmAQP23; Figure S9: 3D models for OmAQP15; Figure S10: 3D models for OmAQP97; Figure S11: 3D models for OmAQP91; Table S1: Transcripts containing full-length ORFs coding for aquaporin proteins recovered from the transcriptomes of midgut and salivary glands of *Ornithodoros moubata*; Table S2: Tick orthologues of the OmAQPs retrieved by BLASTp searching of the NCBInr database restricted to ticks and the Uniprot database restricted to arthropoda; Table S3. Three-dimensional Homology modelling of OmAQPs using Phyre2 and SWISS-MODEL. Templates used and model quality estimation; Table S4: Structural, physicochemical and epitope predictions for OmAQP90; Table S5: Structural, physicochemical and epitope predictions for Om20812; Table S6: Structural, physicochemical and epitope predictions for Om22982; Table S7: Structural, physicochemical and epitope predictions for OmAQP23; Table S8: Structural, physicochemical and epitope predictions for OmAQP15; Table S9: Structural, physicochemical and epitope predictions for OmAQP97; Table S10: Structural, physicochemical and epitope predictions for OmAQP91; Table S11: Physicochemical, allergenic and toxic properties of the selected antigenic peptides.
