# Supplementary material for: First Data on Ornithodoros moubata Aquaporins: Structural, Phylogenetic and Immunogenic Characterisation as Vaccine Targets
Source: Pathogens. 2022 Jun 16;11(6):694. doi: 10.3390/pathogens11060694 (PMC9227307; doi:10.3390/pathogens11060694)
Supplement: Supplementary file 1 [file pathogens-11-00694-s001.zip › Figure S1.pdf]

Figure S1. Nucleotide (**a**) and amino acid (**b**) sequences of the seven *Ornithodoros moubata* AQPs amplified by PCR. (**c**) Nucleotide sequence alignments of the amplified *O. moubata* AQPs with each corresponding transcript as obtained by RNA-seq.

a

```
>OmAQP90
ATGAAGGTGTACATTCGGAGTCTGCTTCTAAGAGAACTCATCAATGAGTTCATTGGAACGATGCTGCTCA
TTCTAATCGGTGACTCCATCATGGCCATTATCATCGCGGGTCGTCAGGAAGAGAATGGTCATGTGATCGG
TCCCCTCGGATGGGGTACAGCCATCTTCTGCGCAGTCTTCGTGGCGGGAGGAGTGTCAGCCCACATCAAC
CCTGCTGTGACGCTGGCCTTCGCTTCCGTCAAAGAGTTTCCTCTCAAGAAAGTGCCCTTATTTTGGCTCG
CCCAGTACTTGGGTGCCTTTGTGGGTGCCGTGCTCGTCTATGTCATCTATAGAGATGCCATCAACATGTT
TGACGGCGGGGTTCGTTCCGTGGTCGGACCCACTGGTACTGCTTCCATATTCTCGACGTATCCACGAGAG
GGCGTCTCTACGCTCACCTGCTTCTTCGACCAGGTCATTTCCACGGCCGTCCTCACGCTCACGGTTGCAG
CAATAGTGGACGAGCGAAACTTTGCCGTCCCCAAAGCCCTCGTTCCTCTCATGCTTGGCATGTTGATCGT
TGCCGAGATTTTTGCCTTCTCCTATAACTGCATGGCTGCTCTGAATCCGGCCCGAGACATTGGACCGCGG
GTGTTCACCGCTGTCGCTGGATGGGGTTCGGAGGTCTTCAGTTTTCGCAACTACCAGTGGGTGTGGGTGC
CCATCTTTGGTCCTCACATTGGGGCCATTGTCGGGGTATGGATATACAAGCTCTGCATAGGGGACCATTG
GCCAATAGAGACGACGCCTGCACTGAAACAAGTACTTTCATCATCGAATGACAAGAGCGGACCTGCTGCG
GAGCCCAAAGAGACAACCAACATCTAG

>Om20812
ATGAAGATACAGAGCACATTCGTCAGGGAACTCATCAACGAATTTCTCGGAACATTTACGCTCATAGTAT
TTGGAGATTCTGTCATGGCCATAATCACTGCAGGACGGCAGGAACACAATGCAGGAATCATCGGCCCTGT
AGGTTGGGGTACTGCTATCTTTTGCGCCGTCACTGTGTCTGGGGGAGTTTCAGCCCACTTAAACCCAGCA
GTAACGCTAGCGTTGGCGTCCGTGAAGAAGTTTCCGATCAAGAAAGTTCCGCTCTTCTTCGTGGCGCAGT
ACACGGGAGCATTCTTTGCCGCTGCGGTTGTTTTTGCAATCTACAGGGACGCCATTGATGCTGTGGACAG
CGGGGTGCGTTCAGTACTGGGTCCAACTGGTACTGCTCCCATATTCGCCACGTATCCCCGTGAGGGAATT
TCCACGCTCACTTGCTTCATTGATCAGGTTGTTGCAACAGGCTGCTTGGTACTGACTGCTGAAGCGATCA
CCGACCCGCGCAACTTCGGAGGACTGCCGAAGCCCTTTTACCCCCTGGCACTGGGCCTCATGATCAGCGC
CGAGATTTTTGCTTTTTCCTACAACTGCATGGCTCCTCTGAACCCGGCTCGAGATCTCGGTCCTAGGGTA
TTCACAGCAATTGCTGGTTGGGGCATGGAGGTTTTCAGTGTGCGCGACTATGTGTGGATGTTTGTGCCCA
TTTTCGCCCCTCATGTTGGAGGCGTGCTTGGTGCGTGGACTTACAAAGTCTGCGTAGAAGACAATTGGCC
AGAAGAGGAACCTACCGTCATTCTTCCCGTCAGCACGAGTAAGAATTCAGAGCTAGGCCCAACTTGGCTG
TGA

>Om22982
ATGAAGATACAGAGCACATTCGTCAGGGAACTCATCAACGAATTTCTCGGAACATTTACGCTCATAGTAT
TTGGAGATTCTGTCATGGCCATAATCATTGCAGGACGGCAGGAACACAATGCAGGAATCATCGGCCCTGT
AGGTTGGGGTACTGCTATCTTTTGCGCCGTCACTGTGTCTGGGGGAGTTTCAGCCCACTTAAACCCAGCA
GTAACGCTAGCGTTGGCGTCCGTGAAGAAGTTTCCGATCAAGAAAGTTCCGCTCTTCTTCGTGGCGCAGT
ACACGGGAGCATTCTTTGCCGCTGCGGTTGTTTTTGCAATATACAGGGACGCCATTGATGCTGTGGACAG
CGGGGTGCGTTCAGTACTGGGTCCAACTGGTACTGCTCCCATATTCGCCACGTATCCCCGTGAGGGAATT
TCCACGCTCACTTGCTTCATTGATCAGGTTGTTACAACAGGCTGCTTGGTACTGACTGCTGAAGCGATCA
CCGACCCGCGCAACTTCGGAGGACTGCCGAAGCCCTTTTACCCCCTGGCACTGGGCCTCATGATCAGCGC
CGAGATTTTTGCTTTTTCCTACAACTGCATGGCTCCTCTGAACCCGGCTCGAGATCTCGGTCCTAGGGTA
TTCACAGCAATTGCTGGTTGGGGCATGGAGGTTTTCAGTGTGCGCGACTATGTGTGGATGTTTGTGCCCA
TTTTCGCCCCTCATGTTGGAGGCGTGCTTGGTGCGTGGACTTACAAAGTCTGCGTAGAAGACAATTGGCC
AGAAGAGGAACCTACCGTCATTCTTCCCGTCAGCACGAGTAAGAATTCAGAGCTAGGTACGACTGCGTCC
GCGGAAATGAATATTTTATTCAGCTGCGATAGTTGTGTATAG
```

>OmAQP91
ATGGGCCGTGTTCGCCAATTCCGCTCAATATTTGGCATCGACGAGCTGTCGGCTACGAGCACGCTCTGGA
AATCAATGATCGCCGAATACATCGGCACGGCCATGCTAGTTTTCATAGCATGCGGAACATGCACCAACTG
GGGTCGAGGGGGAGAACCCAGCATCGCTCAGATTGCGCTCAGTTTCGCTTTCATCGTGGCAACCATGGCT
CAAGCCCTGGGTCATGTCAGCGGAGCGCACATCAACCCAGCGGTGACGATGGGAATGCTGTGCGTCGGCC
ATATCTCCATTCTCCGAGCCTTCTTCTATATCTGCAGTCAGCTCATTGGAGGTATCACTGGAGCAGCCGT
ACTGAGGGCCGTGACACCAGAAGAGCGCCAGGGACTCCTGGGAGGCACGGCACTTTCGGAGGGAGTGACA
CCGTTTCAAGGCGTCGTAGTCGAGCTCGTGATCACTTTCATTCTCGTCTTGACCGTCTTTGCCGTTTGCG
ACCAGAACAGGCTGGACGTCCTGGGATCTCCTCCGCTGGCCATCGGCCTCGCGGTTGGTGCCGGTCATCT
TATGGTGATTGGCTACACAGGAGCCAGCATGAACACAGCCCGTACATTCGGCCCAGCTGTAATCTCAGGA
GCCTTCGATGACCATTGGGTGTACTGGCTTGGCCCCATTGGTGGTGGTATTCTGGCTGGCTTTGTCTACC
AGTACGTCCTCTCTGCTCCACCAATATCCTTCGAAGCTCTGGATAAGGCCCAGCTGGCGTGTCTGGCAAG
GTTTCACAACAAGGAAGCCGTCGAGGACCGCACCACGGCCATCTGA

>OmAQP97
ATGGCAAACCCACCGTTCCCTTTCATGAAGAGATTTGTCATCAAGAGCCCAGTGTTGCGAGAGTTTCTGG
CGGAGTTCTTCGGCACTTTTATCCTTTATATTTTCGGCGGTGCATCGTTCGCTCACTACATTTTCAGCGG
TCAGAAAGATGTGTTGGCGGTGACCTTCTGCTGGGGCATGGGAGTCATGCTCGGCATTCAAACCGGCGCT
GGAGTTTCCGGGGGTCACGTGAATCCCGTCGTAACCACTCCGTTTGCGAGCGTGGGTAGACTTCCCTGGA
AGAAAGTACCCCACTATCTCTTAGGTCAACATCTGGGAGCCTTTCTGGCATCTGCTATCCTCTACTTCAA
TTATATCGATGCGCTGGACCATTATGATGGAGGTGAAAGACAGATATTTGGCGATAGAGGAACGGGAATA
CTTCTGACCACGTTCCCCAATGAACATGTCCACCTCTCCATTTGCATATTCGACACGATCATCTGCTCAG
GCTTGCTCATGTTCACCATCATGGTGATTATCGATAACAAGAACGCGAACACGACATCTGGAATGCACGC
TTTCCTTATCGGGATCATGGTGATGGCGTTGTGCTGGGCCTTCGGCACCAACTGCATGGCTGCCGTCAAC
CCTGCCAGGGATTTCCCACCACGTGTTCTTGCTTCCATTGTGGGATATGGACCCGAAGTGTTCACGTATC
GACACTTCTGGTGGATTCCGATGTTCATTCCCCATGTTGGGGGACTCATCGGCACATGGCTGTACGTGCT
AGCTATTGAGATGCACATAGTGCCAGAAAGGGTACGGACGATAGGACTGAGCGGGAGATGACCAACTTA
TTACCGCTTCCACCGAGGACAGAAAAGACCGGTGTCTAA

>OmAQP23
ATGATTCTGGATAAAGTGAAGATTAAGAGCCTGGCTCTGCGGGAGACGTTCGCCGAACTTCTGGGCACTT
TCGTGCTTGTGCTGTTCGGTACCGGAGTCCTGGCAGCCCTGAAGTTCGACAGAGCTGGAAACATTGGCTA
CGCCGCCGGTTTCTGGGGTTGGGGTTTGGCTCTTGGAATGGGAGTGCTCGTCTCTGGTGGAGCGTCAGGT
GCCCACCTGAACCCGTCTATTACCGTTGCCTTGGCAACCATCGGAAAATGCCAGTGGAGAAAAGTGCCAC
ATTACCTGGTAGCCCAGTACTTAGGTGCCTTCCTTGGAGCTGCCGTGCTCTTCTTGACATACAAGGGTGC
GTTCGACAACTATGACGGTGGTTTTCGTGCGACTACCGGTGTGAACGGAACGGCGGATGTGTTCGCTTCA
TATCCCAGAGACTTCGTTGGCACTGGGAACTGCTTTTTGGACCAGGTCGTCGGGACTGGTCTCTTTGTGA
TGTGCATCGTGGCTATCACAGACAACCACAACATGGCAGTGCCTCAAGGTCTGCAACCTCTGTTGATCGG
CCTTTCTTTGGCAACGACTGCTCTCTGCTTCGGCTACAACTGTGGTGCTCCCCTCAACCCAGCCCGAGAT
TTGGGACCACGTATCTTCACTGCCATGGCTGGGTGGGGAACAGAGGTCTTCAGCTTCCGAGACTACAACT
GGTTCTGGGTGCCGGTAGTGGCGCCCCACATCGGAGCCATCGTTGGAGCATGGCTGTACAGACTCCTGGT
TGGCCTCCACTGGCCTCAAGACTCGTACGACATGGAACAGAGGAACAGGGCCAAGGATGGTGGGTATTCC
CTCGAATGA

>OmAQP15
ATGATTCTGGATAAAGTGAAGATTAAGAGCCTGGCTCTGCGGGAGACGTTCGCCGAACTTCTGGGCACTT
ACGTGCTTGTGCTGTTCGGTACCGGAGTCCTGGCAGCCCTGAAGTTCGACAGAGCTGGAAACATTGGCTA
CGCCGCCGGTTTCTGGGGTTGGGGTTTGGCTCTTGGAATGGGAGTGCTCGTCTCTGGTGGAGCGTCAGGT
GCCCACCTGAACCCGTCTATTACCGTTGCCTTGGCAACCATCGGAAAATGCCAGTGGAGAAAAGTGCCAC
ATTACCTGGTAGCCCAGTACTTAGGTGCCTTCCTTGGAGCTGCCGTGCTCTTCTTGACATACAAGGGTGC
GTTCGACAACTATGACGGTGGTTTTCGTGCGACTACCGGTGTGAACGGAACGGCGGATGTGTTCGCTTCA
TATCCCAGAGACTTCGTTGGCACTGGGAACTGCTTTTTGGACCAGGTCGTCGGGACTGGTCTCTTTGTGA
TGTGCATCGTGGCTATCACAGACAACCACAACATGGCAGTGCCTCAAGGTCTGCAACCTCTGTTGATCGG

CCTTTCTTTGGCAACGACTGCTCTCTGCTTCGGCTACAACTGTGGTGCTCCCCTCAACCCAGCCCGAGAT
TTGGGACCACGTATCTTCACTGCCATGGCTGGGTGGGGAACAGAGGTCTTCAGCTTCCGAGACTACAACT
GGTTCTGGGTGCCGGTAGTGGCGCCCCACATCGGAGCCATCGTTGGAACATGGCTGTACAGACTCCTGGT
TGGCCTCCACTGGCCTCAAGACTCGTACGACATGGAACAGAGGAACAGGGCCAAGGATGAATACATGCCA
ATCGGAGGTTACGATGCTGCGCCAAAGGAACAACAATCAGCCTCATCTCCGAACGAAAAACAGTCGAAGG
TCTAG

b

>OmAQP90
MKVYIRSLLLRELINEFIGTMLLILIGDSIMAIIIAGRQEENGHVIGPLGWGTAIFCAVFVAGGVSAHIN
PAVTLAFASVKEFPLKKVPLFWLAQYLGAFVGAVLVYVIYRDAINMFDGGVRSVVGPTGTASIFSTYPRE
GVSTLTCFFDQVISTAVLTLTVAAIVDERNFAVPKALVPLMLGMLIVAEIFAFSYNCMAALNPARDIGPR
VFTAVAGWGSEVFSFRNYQWVWVPIFGPHIGAIVGVWIYKLCIGDHWPIETTPALKQVLSSSNDKSGPAA
EPKETTNI

>Om20812(MBZ3958194)
MKIQSTFVRELINEFLGTFTLIVFGDSVMAIITAGRQEHNAGIIGPVGWGTAIFCAVTVSGGVSAHLNPA
VTLALASVKKFPIKKVPLFFVAQYTGAFFAAAVVFAIYRDAIDAVDSGVRSVLGPTGTAPIFATYPREGI
STLTCFIDQVVATGCLVLTAEAITDPRNFGGLPKPFYPLALGLMISAEIFAFSYNCMAPLNPARDLGPRV
FTAIAGWGMEVFSVRDYVWMFVPIFAPHVGGVLGAWTYKVCVEDNWPEEEPTVILPVSTSKNSELGPTWL

>Om22982(MBZ3960076)
MKIQSTFVRELINEFLGTFTLIVFGDSVMAIIIAGRQEHNAGIIGPVGWGTAIFCAVTVSGGVSAHLNPA
VTLALASVKKFPIKKVPLFFVAQYTGAFFAAAVVFAIYRDAIDAVDSGVRSVLGPTGTAPIFATYPREGI
STLTCFIDQVVTTGCLVLTAEAITDPRNFGGLPKPFYPLALGLMISAEIFAFSYNCMAPLNPARDLGPRV
FTAIAGWGMEVFSVRDYVWMFVPIFAPHVGGVLGAWTYKVCVEDNWPEEEPTVILPVSTSKNSELGTTAS
AEMNILFSCDSCV

>OmAQP91(A0A1Z5L1C7)
MGRVRQFRSIFGIDELSATSTLWKSMIAEYIGTAMLVFIACGTCTNWGRGGEPSIAQIALSFAFIVATMA
QALGHVSGAHINPAVTMGMLCVGHISILRAFFYICSQLIGGITGAAVLRAVTPEERQGLLGGTALSEGVT
PFQGVVVELVITFILVLTVFAVCDQNRLDVLGSPPLAIGLAVGAGHLMVIGYTGASMNTARTFGPAVISG
AFDDHWVYWLGPIGGGILAGFVYQYVLSAPPISFEALDKAQLACLARFHNKEAVEDRTTAI

>OmAQP97(A0A1Z5L6U6)
MANPPFPFMKRFVIKSPVLREFLAEFFGTFILYIFGGASFAHYIFSGQKDVLAVTFCWGMGVMLGIQTGA
GVSGGHVNPVVTTPFASVGRLPWKKVPHYLLGQHLGAFLASAILYFNYIDALDHYDGGERQIFGDRGTGI
LLTTFPNEHVHLSICIFDTIICSGLLMFTIMVIIDNKNANTTSGMHAFLIGIMVMALCWAFGTNCMAAVN
PARDFPPRVLASIVGYGPEVFTYRHFWWIPMFIPHVGGLIGTWLYVLAIEMHIVPERVRDDRTEREMTNL
LPLPPRTEKTGV

>OmAQP23(A0A1Z5L547)
MILDKVKIKSLALRETFAELLGTFVLVLFGTGVLAALKFDRAGNIGYAAGFWGWGLALGMGVLVSGGASG
AHLNPSITVALATIGKCQWRKVPHYLVAQYLGAFLGAAVLFLTYKGAFDNYDGGFRATTGVNGTADVFAS
YPRDFVGTGNCFLDQVVGTGLFVMCIVAITDNHNMAVPQGLQPLLIGLSLATTALCFGYNCGAPLNPARD
LGPRIFTAMAGWGTEVFSFRDYNWFWVPVVAPHIGAIVGAWLYRLLVGLHWPQDSYDMEQRNRAKDGGYS
LE

>OmAQP15(A0A1Z5KVQ3)
MILDKVKIKSLALRETFAELLGTYVLVLFGTGVLAALKFDRAGNIGYAAGFWGWGLALGMGVLVSGGASG
AHLNPSITVALATIGKCQWRKVPHYLVAQYLGAFLGAAVLFLTYKGAFDNYDGGFRATTGVNGTADVFAS
YPRDFVGTGNCFLDQVVGTGLFVMCIVAITDNHNMAVPQGLQPLLIGLSLATTALCFGYNCGAPLNPARD

LGPRIFTAMAGWGTEVFSFRDYNWFWVPVVAPHIGAIVGTWLYRLLVGLHWPQDSYDMEQRNRAKDEYMP
IGGYDAAPKEQQSASSPNEKQSKV

c

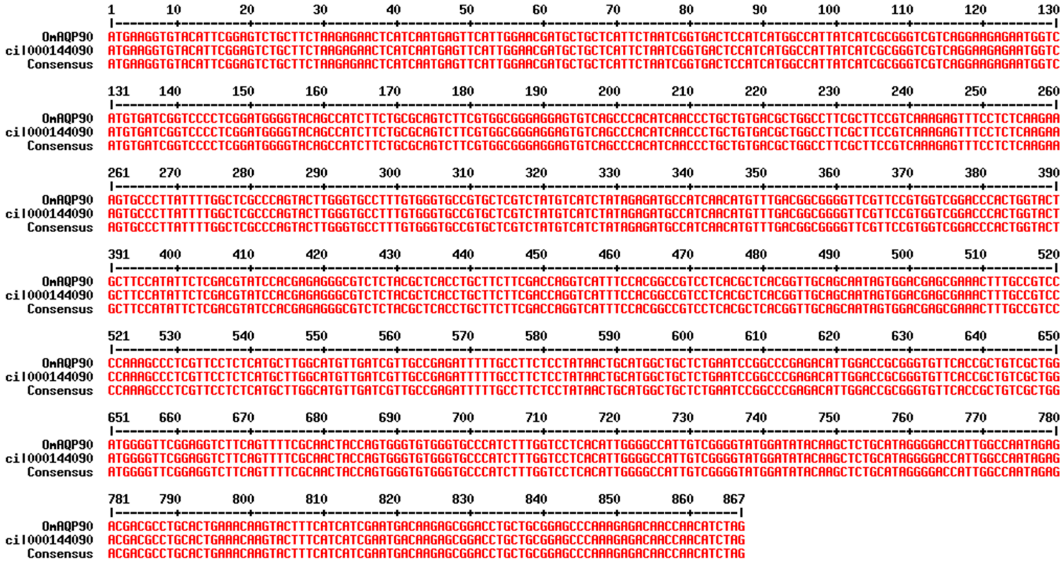

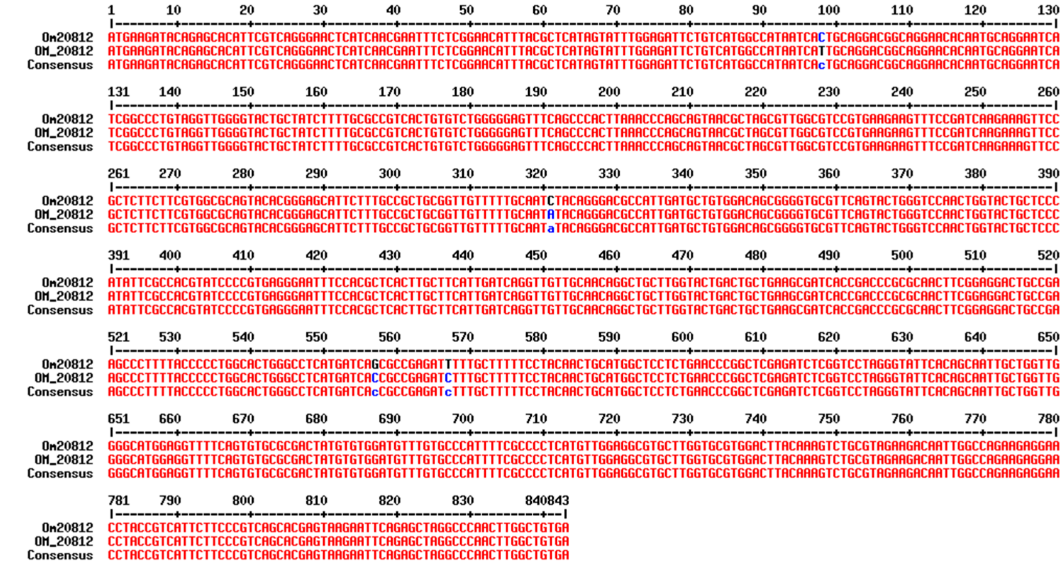

```
           1         10        20        30        40        50        60        70        80        90        100       110       120       130
Om22982,   ATGAAGATACAGAGCACATTCGTCAGGGAACTCATCAACGAATTTCTCGGAACATTTACGCTCATAGTATTTGGAGATTCTGTCATGGCCATAATCATTGCAGGACGGCAGGAACACAATGCAGGAATCA
OM_22982   ATGAAGATACAGAGCACATTCGTCAGGGAACTCATCAACGAATTTCTCGGAACATTTACGCTCATAGTATTTGGAGATTCTGTCATGGCCATAATCATTGCAGGACGGCAGGAACACAATGCAGGAATCA
Consensus  ATGAAGATACAGAGCACATTCGTCAGGGAACTCATCAACGAATTTCTCGGAACATTTACGCTCATAGTATTTGGAGATTCTGTCATGGCCATAATCATTGCAGGACGGCAGGAACACAATGCAGGAATCA

           131       140       150       160       170       180       190       200       210       220       230       240       250       260
Om22982,   TCGGCCCTGTAGGTTGGGGTACTGCTATCTTTTGCGCCGTCACTGTGTCTGGGGGAGTTTCAGCCCACTTAAACCCAGCAGTAACGCTAGCGTTGGCGTCCGTGAAGAAGTTTCCGATCAAGAAAGTTCC
OM_22982   TCGGCCCTGTAGGTTGGGGTACTGCTATCTTTTGCGCCGTCACTGTGTCTGGGGGAGTTTCAGCCCACTTAAACCCAGCAGTAACGCTAGCGTTGGCGTCCGTGAAGAAGTTTCCCATCAAGAAAGTTCC
Consensus  TCGGCCCTGTAGGTTGGGGTACTGCTATCTTTTGCGCCGTCACTGTGTCTGGGGGAGTTTCAGCCCACTTAAACCCAGCAGTAACGCTAGCGTTGGCGTCCGTGAAGAAGTTTCCcATCAAGAAAGTTCC

           261       270       280       290       300       310       320       330       340       350       360       370       380       390
Om22982,   GCTCTTCTTCGTGGCGCAGTACACGGGAGCATTCTTTGCCGCTGCGGTTGTTTTTGCAATATACAGGGACGCCATTGATGCTGTGGACAGCGGGGTGCGTTCAGTACTGGGTCCAACTGGTACTGCTCCC
OM_22982   GCTCTTCTTCGTGGCGCAGTACACGGGAGCATTCTTTGCCGCTGCGGTTGTTTTTGCAATATACAGGGACGCCATTGATGCTGTGGACAGCGGGGTGCGTTCAGTACTGGGTCCAACTGGTACTGCTCCC
Consensus  GCTCTTCTTCGTGGCGCAGTACACGGGAGCATTCTTTGCCGCTGCGGTTGTTTTTGCAATATACAGGGACGCCATTGATGCTGTGGACAGCGGGGTGCGTTCAGTACTGGGTCCAACTGGTACTGCTCCC

           391       400       410       420       430       440       450       460       470       480       490       500       510       520
Om22982,   ATATTCGCCACGTATCCCCGTGAGGGAATTTCCACGCTCACTTGCTTCATTGATCAGGTTGTTACAACAGGCTGCTTGGTACTGACTGCTGAAGCGATCACCGACCCGCGCAACTTCGGAGGACTGCCGA
OM_22982   ATATTCGCCACGTATCCCCGTGAGGGAATTTCCACGCTCACTTGCTTCATTGATCAGGTTGTTGCAACAGGCTGCTTGGTACTGACTGCTGAAGCGATCACCGACCCGCGCAACTTCGGAGGACTGCCGA
Consensus  ATATTCGCCACGTATCCCCGTGAGGGAATTTCCACGCTCACTTGCTTCATTGATCAGGTTGTTaCAACAGGCTGCTTGGTACTGACTGCTGAAGCGATCACCGACCCGCGCAACTTCGGAGGACTGCCGA

           521       530       540       550       560       570       580       590       600       610       620       630       640       650
Om22982,   AGCCCTTTTACCCCCTGGCACTGGGCCTCATGATCAGCGCCGAGATTTTTGCTTTTTCCTACAACTGCATGGCTCCTCTGAACCCGGCTCGAGATCTCGGTCCTAGGGTATTCACAGCAATTGCTGGTTG
OM_22982   AGCCCTTTTACCCCCTGGCACTGGGCCTCATGATCACCGCCGAGATCTTTGCTTTTTCCTACAACTGCATGGCTCCTCTGAACCCGGCTCGAGATCTCGGTCCTAGGGTATTCACAGCAATTGCTGGTTG
Consensus  AGCCCTTTTACCCCCTGGCACTGGGCCTCATGATCAcCGCCGAGATcTTTGCTTTTTCCTACAACTGCATGGCTCCTCTGAACCCGGCTCGAGATCTCGGTCCTAGGGTATTCACAGCAATTGCTGGTTG

           651       660       670       680       690       700       710       720       730       740       750       760       770       780
Om22982,   GGGCATGGAGGTTTTCAGTGTGCGCGACTATGTGTGGATGTTTGTGCCCATTTTCGCCCCTCATGTTGGAGGCGTGCTTGGTGCGTGGACTTACAAAGTCTGCGTAGAAGACAATTGGCCAGAAGAGGAA
OM_22982   GGGCATGGAGGTTTTCAGTGTGCGCGACTATGTGTGGATGTTTGTGCCCATTTTCGCCCCTCATGTTGGAGGCGTGCTTGGTGCGTGGACTTACAAAGTCTGCGTAGAAGACAATTGGCCAGAAGAGGAA
Consensus  GGGCATGGAGGTTTTCAGTGTGCGCGACTATGTGTGGATGTTTGTGCCCATTTTCGCCCCTCATGTTGGAGGCGTGCTTGGTGCGTGGACTTACAAAGTCTGCGTAGAAGACAATTGGCCAGAAGAGGAA

           781       790       800       810       820       830       840       850       860       870       88082
Om22982,   CCTACCGTCATTCTTCCCGTCAGCACGAGTAAGAATTCAGAGCTAGGTACGACTGCGTCCGCGGAAATGAATATTTTATTCAGCTGCGATAGTTGTGTATAG
OM_22982   CCTACCGTCATTCTTCCCGTCAGCACGAGTAAGAATTCAGAGCTAGGTACGACTGCGTCCGCGGAAATGAATATTTTATTCAGCTGCGATAGTTGTGTATAG
Consensus  CCTACCGTCATTCTTCCCGTCAGCACGAGTAAGAATTCAGAGCTAGGTACGACTGCGTCCGCGGAAATGAATATTTTATTCAGCTGCGATAGTTGTGTATAG
```

```
             1         10        20        30        40        50        60        70        80        90        100       110       120       130
OmAQP91      ATGGGCCGTGTTCGCCAATTCCGCTCAATATTTGGCATCGACGAGCTGTCGGCTACGAGCACGCTCTGGAAATCAATGATCGCCGAATACATCGGCACGGCCATGCTAGTTTTCATAGCATGCGGAACAT
ci|000124891 ATGGGCCGTGTTCGCCAATTCCGCTCAATATTTGGCATCGACGAGCTGTCGGCTACGAGCATGCTCTGGAAATCAATGATCGCCGAATACATCGGCACGGCCATGCTAGTTTTCATAGCATGCGGAACAT
Consensus    ATGGGCCGTGTTCGCCAATTCCGCTCAATATTTGGCATCGACGAGCTGTCGGCTACGAGCAcGCTCTGGAAATCAATGATCGCCGAATACATCGGCACGGCCATGCTAGTTTTCATAGCATGCGGAACAT

             131       140       150       160       170       180       190       200       210       220       230       240       250       260
OmAQP91      GCACCAACTGGGGTCGAGGGGGAGAACCCAGCATCGCTCAGATTGCGCTCAGTTTCGCTTTCATCGTGGCAACCATGGCTCAAGCCCTGGGTCATGTCAGCGGAGCGCACATCAACCCAGCGGTGACGAT
ci|000124891 GCACCAACTGGGGTCGAGGGGGAGAACCCAGCATCGCTCAGATTGCGCTCAGTTTCGCTTTCATCGTGGCAACCATGGCTCAAGCCCTGGGTCATGTCAGCGGAGCGCACATCAACCCAGCGGTGACGAT
Consensus    GCACCAACTGGGGTCGAGGGGGAGAACCCAGCATCGCTCAGATTGCGCTCAGTTTCGCTTTCATCGTGGCAACCATGGCTCAAGCCCTGGGTCATGTCAGCGGAGCGCACATCAACCCAGCGGTGACGAT

             261       270       280       290       300       310       320       330       340       350       360       370       380       390
OmAQP91      GGGAATGCTGTGCGTCGGCCATATCTCCATTCTCCGAGCCTTCTTCTATATCTGCAGTCAGCTCATTGGAGGTATCACTGGAGCAGCCGTACTGAGGGCCGTGACACCAGAAGAGCGCCAGGGACTCCTG
ci|000124891 GGGAATGCTGTGCGTCGGCCATATCTCCATTCTCCGAGCCTTCTTCTATATCTGCAGTCAGCTCATTGGAGGTATCACTGGAGCAGCCGTACTGAGGGCCGTGACACCAGAAGAGCGCCAGGGACTCCTG
Consensus    GGGAATGCTGTGCGTCGGCCATATCTCCATTCTCCGAGCCTTCTTCTATATCTGCAGTCAGCTCATTGGAGGTATCACTGGAGCAGCCGTACTGAGGGCCGTGACACCAGAAGAGCGCCAGGGACTCCTG

             391       400       410       420       430       440       450       460       470       480       490       500       510       520
OmAQP91      GGAGGCACGGCACTTTCGGAGGGAGTGACACCGTTTCAAGGCGTCGTAGTCGAGCTCGTGATCACTTTCATTCTCGTCTTGACCGTCTTTGCCGTTTGCGACCAGAACAGGCTGGACGTCCTGGGATCTC
ci|000124891 GGAGGCACGGCACTTTCGGAGGGAGTGACACCGTTTCAAGGCGTCGTAGTCGAGCTCGTGATCACTTTCATTCTCGTCTTGACCGTCTTTGCCGTTTGCGACCAGAACAGGCTGGACGTCCTGGGATCTC
Consensus    GGAGGCACGGCACTTTCGGAGGGAGTGACACCGTTTCAAGGCGTCGTAGTCGAGCTCGTGATCACTTTCATTCTCGTCTTGACCGTCTTTGCCGTTTGCGACCAGAACAGGCTGGACGTCCTGGGATCTC

             521       530       540       550       560       570       580       590       600       610       620       630       640       650
OmAQP91      CTCCGCTGGCCATCGGCCTCGCGGTTGGTGCCGGTCATCTTATGGTGATTGGCTACACAGGAGCCAGCATGAACACAGCCCGTACATTCGGCCCAGCTGTAATCTCAGGAGCCTTCGATGACCATTGGGT
ci|000124891 CTCCGCTGGCCATCGGCCTCGCGGTTGGTGCCGGTCATCTTATGGCGATTGGCTACACAGGAGCCAGCATGAACACAGCCCGTACATTCGGCCCAGCTGTAATCTCAGGAGCCTTCGATGACCATTGGGT
Consensus    CTCCGCTGGCCATCGGCCTCGCGGTTGGTGCCGGTCATCTTATGGcGATTGGCTACACAGGAGCCAGCATGAACACAGCCCGTACATTCGGCCCAGCTGTAATCTCAGGAGCCTTCGATGACCATTGGGT

             651       660       670       680       690       700       710       720       730       740       750       760       770       780
OmAQP91      GTACTGGCTTGGCCCCATTGGTGGTGGTATTCTGGCTGGCTTTGTCTACCAGTACGTCCTCTCTGCTCCACCAATATCCTTCGAAGCTCTGGATAAGGCCCAGCTGGCGTGTCTGGCAAGGTTTCACAAC
ci|000124891 GTACTGGCTTGGCCCCATTGGTGGTGGTATTCTGGCTGGCTTTGTCTACCAGTACGTCCTCTCTGCTCCACCAATATCCTTCGAAGCTCTGGATAAGGCCCAGCTGGCGTGTCTGGCAAGGTTTCACAAC
Consensus    GTACTGGCTTGGCCCCATTGGTGGTGGTATTCTGGCTGGCTTTGTCTACCAGTACGTCCTCTCTGCTCCACCAATATCCTTCGAAGCTCTGGATAAGGCCCAGCTGGCGTGTCTGGCAAGGTTTCACAAC

             781       790       800       810   816
OmAQP91      AAGGAAGCCGTCGAGGACCGCACCACGGCCATCTGA
ci|000124891 AAGGAAGCCGTCGAGGACCGCACCACGGCCATCTGA
Consensus    AAGGAAGCCGTCGAGGACCGCACCACGGCCATCTGA
```

```
           1       10        20        30        40        50        60        70        80        90        100       110       120       130
OmAQP97    ATGGCAAACCCACCGTTCCCTTTCATGAAGAGATTTGTCATCAAGAGCCCAGTGTTGCGAGAGTTTCTGGCGGAGTTCTTCGGCACTTTTATCCTTTATATTTTCGGCGGTGCATCGTTCGCTCACTACA
ci|000113997 ATGGCAAACCCACCGTTCCCTTTCATGAAGAGATTTGTCATCAAGAGCCCAGTGTTGCGAGAGTTTCTGGCGGAGTTCTTCGGCACTTTTATCCTTTATATTTTCGGCGGTGCATCGTTCGCTCACTACA
Consensus  ATGGCAAACCCACCGTTCCCTTTCATGAAGAGATTTGTCATCAAGAGCCCAGTGTTGCGAGAGTTTCTGGCGGAGTTCTTCGGCACTTTTATCCTTTATATTTTCGGCGGTGCATCGTTCGCTCACTACA

           131     140       150       160       170       180       190       200       210       220       230       240       250       260
OmAQP97    TTTTCAGCGGTCAGAAAGATGTGTTGGCGGTGACCTTCTGCTGGGGCATGGGAGTCATGCTCGGCATTCAAACCGGCGCTGGAGTTTCCGGGGGTCACGTGAATCCCGTCGTAACCACTCCGTTTGCGAG
ci|000113997 TTTTCAGCGGTCAGAAAGATGTGTTGGCGGTGACCTTCTGCTGGGGCATGGGAGTCATGCTCGGCATTCAAACCGGCGCTGGAGTTTCCGGGGGTCACGTGAATCCCGTCGTAACCACTTCGTTTGCGAG
Consensus  TTTTCAGCGGTCAGAAAGATGTGTTGGCGGTGACCTTCTGCTGGGGCATGGGAGTCATGCTCGGCATTCAAACCGGCGCTGGAGTTTCCGGGGGTCACGTGAATCCCGTCGTAACCACTcCGTTTGCGAG

           261     270       280       290       300       310       320       330       340       350       360       370       380       390
OmAQP97    CGTGGGTAGACTTCCCTGGAAGAAAGTACCCCACTATCTCTTAGGTCAACATCTGGGAGCCTTTCTGGCATCTGCTATCCTCTACTTCAATTATATCGATGCGCTGGACCATTATGATGGAGGTGAAAGA
ci|000113997 CGTGGGTAGACTTCCCTGGAAGAAAGTACCCCACTATCTCTTAGGTCAACATCTGGGAGCCTTTCTGGCATCTGCTATCCTCTACTTCAATTATATCGATGCGCTGGACCATTATGATGGAGGTGAAAGA
Consensus  CGTGGGTAGACTTCCCTGGAAGAAAGTACCCCACTATCTCTTAGGTCAACATCTGGGAGCCTTTCTGGCATCTGCTATCCTCTACTTCAATTATATCGATGCGCTGGACCATTATGATGGAGGTGAAAGA

           391     400       410       420       430       440       450       460       470       480       490       500       510       520
OmAQP97    CAGATATTTGGCGATAGAGGAACGGGAATACTTCTGACCACGTTCCCCAATGAACATGTCCACCTCTCCATTTGCATATTCGACACGATCATCTGCTCAGGCTTGCTCATGTTCACCATCATGGTGATTA
ci|000113997 CAGATATTTGGCGATAGAGGAACGGGAATACTTCTGACCACGTTCCCCAATGAACATGTCCACCTCTCCATTTGCATATTCGACACGATCATCTGCTCAGGCTTGCTCATGTTCACCATCATGGTGATTA
Consensus  CAGATATTTGGCGATAGAGGAACGGGAATACTTCTGACCACGTTCCCCAATGAACATGTCCACCTCTCCATTTGCATATTCGACACGATCATCTGCTCAGGCTTGCTCATGTTCACCATCATGGTGATTA

           521     530       540       550       560       570       580       590       600       610       620       630       640       650
OmAQP97    TCGATAACAAGAACGCGAACACGACATCTGGAATGCACGCTTTCCTTATCGGGATCATGGTGATGGCGTTGTGCTGGGCCTTCGGCACCAACTGCATGGCTGCCGTCAACCCTGCCAGGGATTTCCCACC
ci|000113997 TCGATAACAAGAACGCGAACACGACATCTGGAATGCACGCTTTCCTTTTCGGGATCATGGTGATGGCGTTGTGCTGGGCCTTCGGCACCAACTGCATGGCTGCCGTCAACCCTGCCAGGGATTTCCCACC
Consensus  TCGATAACAAGAACGCGAACACGACATCTGGAATGCACGCTTTCCTTaTCGGGATCATGGTGATGGCGTTGTGCTGGGCCTTCGGCACCAACTGCATGGCTGCCGTCAACCCTGCCAGGGATTTCCCACC

           651     660       670       680       690       700       710       720       730       740       750       760       770       780
OmAQP97    ACGTGTTCTTGCTTCCATTGTGGGATATGGACCCGAAGTGTTCACGTATCGACACTTCTGGTGGATTCCGATGTTCATTCCCCATGTTGGGGGACTCATCGGCACATGGCTGTACGTGCTAGCTATTGAG
ci|000113997 ACGTGTTCTTGCTTCCATTGTGGGATATGGACCCGAAGTGTTCACGTATCGACACTTCTGGTGGATTCCGATGTTCATTCCCCATGTTGGGGGACTCATCGGCACATGGCTGTACGTGCTAGCTATTGAG
Consensus  ACGTGTTCTTGCTTCCATTGTGGGATATGGACCCGAAGTGTTCACGTATCGACACTTCTGGTGGATTCCGATGTTCATTCCCCATGTTGGGGGACTCATCGGCACATGGCTGTACGTGCTAGCTATTGAG

           781     790       800       810       820       830       840       850       860       870       879
OmAQP97    ATGCACATAGTGCCAGAAAGGGTACGGGACGATAGGACTGAGCGGGAGATGACCAACTTATTACCGCTTCCACCGAGGACAGAAAAGACCGGTGTCTAA
ci|000113997 ATGCACATAGTGCCAGAAAGGGTACGGGACGATAGGACTGAGCGGGAGATGACCAACTTATTACCGCTTCCATCGAGGACAGAAAAGACCGGTGTCTAA
Consensus  ATGCACATAGTGCCAGAAAGGGTACGGGACGATAGGACTGAGCGGGAGATGACCAACTTATTACCGCTTCCAcCGAGGACAGAAAAGACCGGTGTCTAA
```

```
           1       10        20        30        40        50        60        70        80        90        100       110       120       130
OmAQP23    ATGATTCTGGATAAAGTGAAGATTAAGAGCCTGGCTCTGCGGGAGACGTTCGCCGAACTTCTGGGCACTTTCGTGCTTGTGCTGTTCGGTACCGGAGTCCTGGCAGCCCTGAAGTTCGACAGAGCTGGAA
ci|000114723 ATGATTCTGGATAAAGTGAAGATTAAGAGCCTGGCTCTGCGGGAGACGTTCGCCGAACTTCTGGGCACTTTCGTGCTTGTGCTGTTCGGTACCGGAGTCCTGGCAGCCCTGAAGTTCGACAGAGCTGGAA
Consensus  ATGATTCTGGATAAAGTGAAGATTAAGAGCCTGGCTCTGCGGGAGACGTTCGCCGAACTTCTGGGCACTTTCGTGCTTGTGCTGTTCGGTACCGGAGTCCTGGCAGCCCTGAAGTTCGACAGAGCTGGAA

           131     140       150       160       170       180       190       200       210       220       230       240       250       260
OmAQP23    ACATTGGCTACGCCGCCGGTTTCTGGGGTTGGGGTTTGGCTCTTGGAATGGGAGTGCTCGTCTCTGGTGGAGCGTCAGGTGCCCACCTGAACCCGTCTATTACCGTTGCCTTGGCAACCATCGGAAAATG
ci|000114723 ACATTGGCTACGCCGCCGGTTTCTGGGGTTGGGGTTTGGCTCTTGGAATGGGAGTGCTCGTCTCTGGTGGAGCGTCAGGTGCCCACCTGAACCCGTCTATTACCGTTGCCTTGGCAACCATCGGAAAATG
Consensus  ACATTGGCTACGCCGCCGGTTTCTGGGGTTGGGGTTTGGCTCTTGGAATGGGAGTGCTCGTCTCTGGTGGAGCGTCAGGTGCCCACCTGAACCCGTCTATTACCGTTGCCTTGGCAACCATCGGAAAATG

           261     270       280       290       300       310       320       330       340       350       360       370       380       390
OmAQP23    CCAGTGGAGAAAAGTGCCACATTACCTGGTAGCCCAGTACTTAGGTGCCTTCCTTGGAGCTGCCGTGCTCTTCTTGACATACAAGGGTGCGTTCGACAACTATGACGGTGGTTTTCGTGCGACTACCGGT
ci|000114723 CCAGTGGAGAAAAGTGCCACATTACCTGGTAGCCCAGTACTTAGGTGCCTTCCTTGGAGCTGCCGTGCTCTTCTTGACATACAAGGGTGCGTTCGACAACTATGACGGTGGTTTTCGTGCGACTACCGGT
Consensus  CCAGTGGAGAAAAGTGCCACATTACCTGGTAGCCCAGTACTTAGGTGCCTTCCTTGGAGCTGCCGTGCTCTTCTTGACATACAAGGGTGCGTTCGACAACTATGACGGTGGTTTTCGTGCGACTACCGGT

           391     400       410       420       430       440       450       460       470       480       490       500       510       520
OmAQP23    GTGAACGGAACGGCGGATGTGTTCGCTTCATATCCCAGAGACTTCGTTGGCACTGGGAACTGCTTTTTGGACCAGGTCGTCGGGACTGGTCTCTTTGTGATGTGCATCGTGGCTATCACAGACAACCACA
ci|000114723 GTGAACGGAACGGCGGATGTGTTCGCTTCATATCCCAGAGACTTCGTTGGCACTGGGAACTGCTTTTTGGACCAGGTCGTCGGGACTGGTCTCTTTGTGATGTGCATCGTGGCTATCACAGACAACCACA
Consensus  GTGAACGGAACGGCGGATGTGTTCGCTTCATATCCCAGAGACTTCGTTGGCACTGGGAACTGCTTTTTGGACCAGGTCGTCGGGACTGGTCTCTTTGTGATGTGCATCGTGGCTATCACAGACAACCACA

           521     530       540       550       560       570       580       590       600       610       620       630       640       650
OmAQP23    ACATGGCAGTGCCTCAAGGTCTGCAACCTCTGTTGATCGGCCTTTCTTTGGCAACGACTGCTCTCTGCTTCGGCTACAACTGTGGTGCTCCCCTCAACCCAGCCCGAGATTTGGGACCACGTATCTTCAC
ci|000114723 ACATGGCAGTGCCTCAAGGTCTGCAACCTCTGTTGATCGGCCTTTCTTTGGCAACGACTGCTCTCTGCTTCGGCTACAACTGTGGTGCTCCCCTCAACCCAGCCCGAGATTTGGGACCACGTATCTTCAC
Consensus  ACATGGCAGTGCCTCAAGGTCTGCAACCTCTGTTGATCGGCCTTTCTTTGGCAACGACTGCTCTCTGCTTCGGCTACAACTGTGGTGCTCCCCTCAACCCAGCCCGAGATTTGGGACCACGTATCTTCAC

           651     660       670       680       690       700       710       720       730       740       750       760       770       780
OmAQP23    TGCCATGGCTGGGTGGGGAACAGAGGTCTTCAGCTTCCGAGACTACAACTGGTTCTGGGTGCCGGTAGTGGCGCCCCACATCGGAGCCATCGTTGGAGCATGGCTGTACAGACTCCTGGTTGGCCTCCAC
ci|000114723 TGCCATGGCTGGGTGGGGAACAGAGGTCTTCAGCTTCCGAGACTACAACTGGTTCTGGGTGCCGGTAGTGGCGCCCCACATCGGAGCCATCGTTGGAACATGGCTGTACAGACTCCTGGTTGGCCTCCAC
Consensus  TGCCATGGCTGGGTGGGGAACAGAGGTCTTCAGCTTCCGAGACTACAACTGGTTCTGGGTGCCGGTAGTGGCGCCCCACATCGGAGCCATCGTTGGAaCATGGCTGTACAGACTCCTGGTTGGCCTCCAC

           781     790       800       810       820       830       840       849
OmAQP23    TGGCCTCAAGACTCGTACGACATGGAACAGAGGAACAGGGCCAAGGATGGTGGGTATTCCCTCGAATGA
ci|000114723 TGGCCTCAAGACTCGTACGACATGGAACAGAGGAACAGGGCCAAGGATGGTGGGTATTCCCTCGAATGA
Consensus  TGGCCTCAAGACTCGTACGACATGGAACAGAGGAACAGGGCCAAGGATGGTGGGTATTCCCTCGAATGA
```

```
             1        10        20        30        40        50        60        70        80        90        100       110       120       130
             |--------+---------+---------+---------+---------+---------+---------+---------+---------+---------+---------+---------+---------|
OmAQP15      ATGATTCTGGATAAAGTGAAGATTAAGAGCCTGGCTCTGCGGGAGACGTTCGCCGAACTTCTGGGCACTTACGTGCTTGTGCTGTTCGGTACCGGAGTCCTGGCAGCCCTGAAGTTCGACAGAGCTGGAA
ci|000148315 ATGATTCTGGATAAAGTGAAGATTAAGAGCCTGGCTCTGCGGGAGACGTTCGCCGAACTTCTGGGCACTTTCGTGCTTGTGCTGTTCGGTACCGGAGTCCTGGCAGCCCTGAAGTTCGACAGAGCTGGAA
Consensus    ATGATTCTGGATAAAGTGAAGATTAAGAGCCTGGCTCTGCGGGAGACGTTCGCCGAACTTCTGGGCACTTaCGTGCTTGTGCTGTTCGGTACCGGAGTCCTGGCAGCCCTGAAGTTCGACAGAGCTGGAA

             131      140       150       160       170       180       190       200       210       220       230       240       250       260
             |--------+---------+---------+---------+---------+---------+---------+---------+---------+---------+---------+---------+---------|
OmAQP15      ACATTGGCTACGCCGCCGGTTTCTGGGGTTGGGGTTTGGCTCTTGGAATGGGAGTGCTCGTCTCTGGTGGAGCGTCAGGTGCCCACCTGAACCCGTCTATTACCGTTGCCTTGGCAACCATCGGAAAATG
ci|000148315 ACATTGGCTACGCCGCCGGTTTCTGGGGTTGGGGTTTGGCTCTTGGAATGGGAGTGCTCGTCTCTGGTGGAGCGTCAGGTGCCCACCTGAACCCGTCTATTACCGTTGCCTTGGCAACCATCGGAAAATG
Consensus    ACATTGGCTACGCCGCCGGTTTCTGGGGTTGGGGTTTGGCTCTTGGAATGGGAGTGCTCGTCTCTGGTGGAGCGTCAGGTGCCCACCTGAACCCGTCTATTACCGTTGCCTTGGCAACCATCGGAAAATG

             261      270       280       290       300       310       320       330       340       350       360       370       380       390
             |--------+---------+---------+---------+---------+---------+---------+---------+---------+---------+---------+---------+---------|
OmAQP15      CCAGTGGAGAAAAGTGCCACATTACCTGGTAGCCCAGTACTTAGGTGCCTTCCTTGGAGCTGCCGTGCTCTTCTTGACATACAAGGGTGCGTTCGACAACTATGACGGTGGTTTTCGTGCGACTACCGGT
ci|000148315 CCAGTGGAGAAAAGTGCCACATTACCTGGTAGCCCAGTACTTAGGTGCCTTCCTTGGAGCTGCCGTGCTCTTCTTGACATACAAGGGTGCGTTCGACAACTATGACGGTGGTTTTCGTGCGACTACCGGT
Consensus    CCAGTGGAGAAAAGTGCCACATTACCTGGTAGCCCAGTACTTAGGTGCCTTCCTTGGAGCTGCCGTGCTCTTCTTGACATACAAGGGTGCGTTCGACAACTATGACGGTGGTTTTCGTGCGACTACCGGT

             391      400       410       420       430       440       450       460       470       480       490       500       510       520
             |--------+---------+---------+---------+---------+---------+---------+---------+---------+---------+---------+---------+---------|
OmAQP15      GTGAACGGAACGGCGGATGTGTTCGCTTCATATCCCAGAGACTTCGTTGGCACTGGGAACTGCTTTTTGGACCAGGTCGTCGGGACTGGTCTCTTTGTGATGTGCATCGTGGCTATCACAGACAACCACA
ci|000148315 GTGAACGGAACGGCGGATGTGTTCGCTTCATATCCCAGAGACTTCGTTGGCACTGGGAACTGCTTTTTGGACCAGGTCGTCGGGACTGGTCTCTTTGTGATGTGCATCGTGGCTATCACAGACAACCACA
Consensus    GTGAACGGAACGGCGGATGTGTTCGCTTCATATCCCAGAGACTTCGTTGGCACTGGGAACTGCTTTTTGGACCAGGTCGTCGGGACTGGTCTCTTTGTGATGTGCATCGTGGCTATCACAGACAACCACA

             521      530       540       550       560       570       580       590       600       610       620       630       640       650
             |--------+---------+---------+---------+---------+---------+---------+---------+---------+---------+---------+---------+---------|
OmAQP15      ACATGGCAGTGCCTCAAGGTCTGCAACCTCTGTTGATCGGCCTTTCTTTGGCAACGACTGCTCTCTGCTTCGGCTACAACTGTGGTGCTCCCCTCAACCCAGCCCGAGATTTGGGACCACGTATCTTCAC
ci|000148315 ACATGGCAGTGCCTCAAGGTCTGCAACCTCTGTTGATCGGCCTTTCTTTGGCAACGACTGCTCTCTGCTTCGGCTACAACTGTGGTGCTCCCCTCAACCCAGCCCGAGATTTGGGACCACGTATCTTCAC
Consensus    ACATGGCAGTGCCTCAAGGTCTGCAACCTCTGTTGATCGGCCTTTCTTTGGCAACGACTGCTCTCTGCTTCGGCTACAACTGTGGTGCTCCCCTCAACCCAGCCCGAGATTTGGGACCACGTATCTTCAC

             651      660       670       680       690       700       710       720       730       740       750       760       770       780
             |--------+---------+---------+---------+---------+---------+---------+---------+---------+---------+---------+---------+---------|
OmAQP15      TGCCATGGCTGGGTGGGGAACAGAGGTCTTCAGCTTCCGAGACTACAACTGGTTCTGGGTGCCGGTAGTGGCGCCCCACATCGGAGCCATCGTTGGAACATGGCTGTACAGACTCCTGGTTGGCCTCCAC
ci|000148315 TGCCATGGCTGGGTGGGGAACAGAGGTCTTCAGCTTCCGAGACTACAACTGGTTCTGGGTGCCGGTAGTGGCGCCCCACATCGGAGCCATCGTTGGAACATGGCTGTACAGACTCCTGGTTGGCCTCCAC
Consensus    TGCCATGGCTGGGTGGGGAACAGAGGTCTTCAGCTTCCGAGACTACAACTGGTTCTGGGTGCCGGTAGTGGCGCCCCACATCGGAGCCATCGTTGGAACATGGCTGTACAGACTCCTGGTTGGCCTCCAC

             781      790       800       810       820       830       840       850       860       870       880       890       900       910
             |--------+---------+---------+---------+---------+---------+---------+---------+---------+---------+---------+---------+---------|
OmAQP15      TGGCCTCAAGACTCGTACGACATGGAACAGAGGAACAGGGCCAAGGATGAATACATGCCAATCGGAGGTTACGATGCTGCGCCAAAGGAACAACAATCAGCCTCATCTCCGAACGAAAAACAGTCGAAGG
ci|000148315 TGGCCTCAAGACTCGTACGACATGGAACAGAGGAACAGGGCCAAGGATGAATACATGCCAATCGGAGGTTACGATGCTGCGCCAAAGGAACAACAATCAGCCTCATCTCCGAACGAAAAACAGTCGAAGG
Consensus    TGGCCTCAAGACTCGTACGACATGGAACAGAGGAACAGGGCCAAGGATGAATACATGCCAATCGGAGGTTACGATGCTGCGCCAAAGGAACAACAATCAGCCTCATCTCCGAACGAAAAACAGTCGAAGG

             911 915
             |---|
OmAQP15      TCTAG
ci|000148315 TCTAG
Consensus    TCTAG
```
