# Supplementary material for: First Data on Ornithodoros moubata Aquaporins: Structural, Phylogenetic and Immunogenic Characterisation as Vaccine Targets
Source: Pathogens. 2022 Jun 16;11(6):694. doi: 10.3390/pathogens11060694 (PMC9227307; doi:10.3390/pathogens11060694)

Figure S10. 3D models for OmAQP97. (a) and (b) Side and top view, respectively, of the monomer cartoon showing the position of the two NPA motifs (red spheres) and the aromatic arginine (ar/R) constriction (orange spheres); (c) and (d) side and top view of the tetramer cartoon; (e) monomer mesh and surface model showing the exposure of the 3 antigenic peptides on the extracellular surface of the molecule: OmAQP97_A yellow, OmAQP97_C orange, and OmAQP97_E cyan. (f) side and top view of tetramer surface model showing peptide exposure.

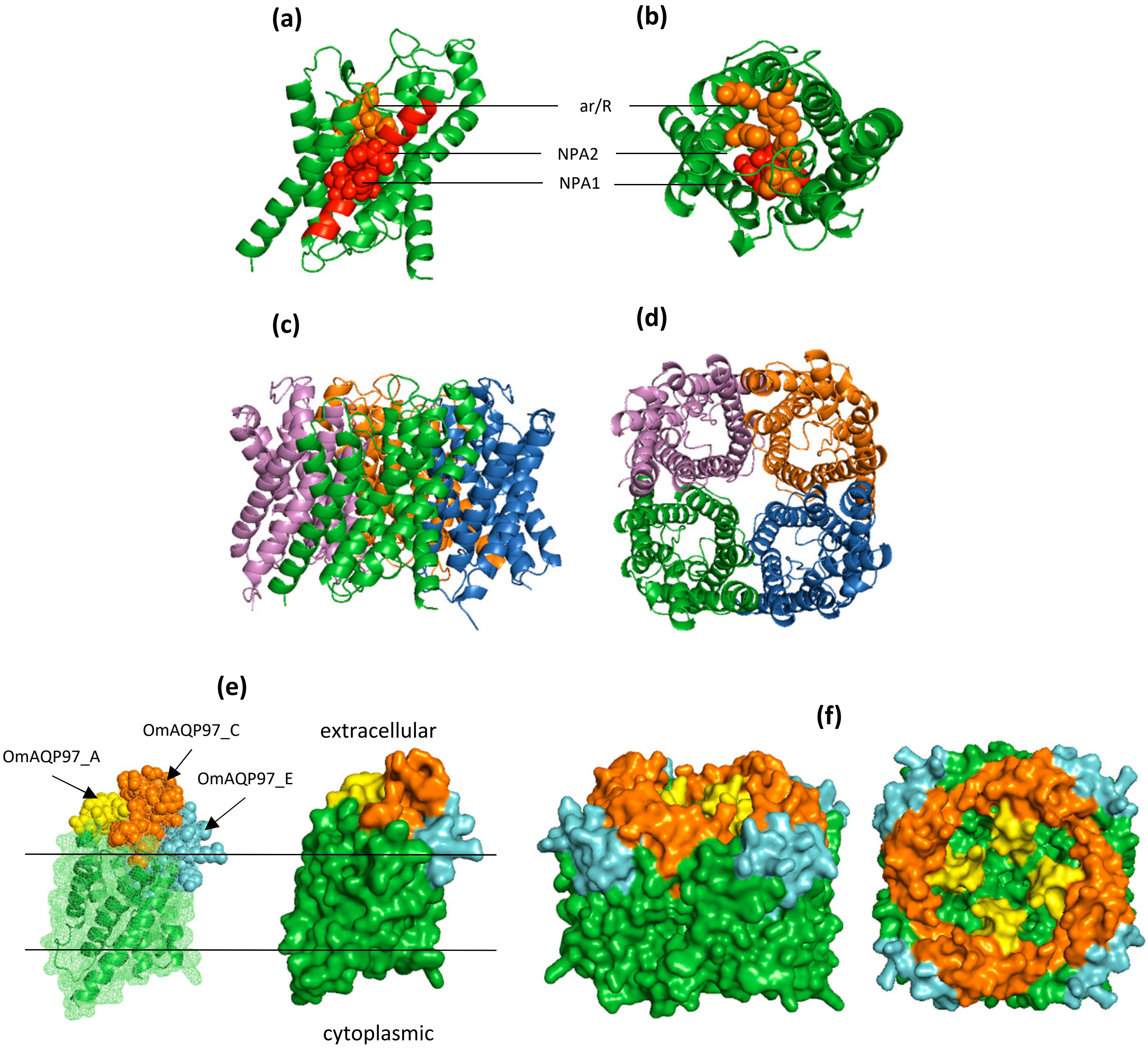

Supplement: Supplementary file 1 [file pathogens-11-00694-s001.zip › Figure S10.pdf]
