# Supplementary material for: First Data on Ornithodoros moubata Aquaporins: Structural, Phylogenetic and Immunogenic Characterisation as Vaccine Targets
Source: Pathogens. 2022 Jun 16;11(6):694. doi: 10.3390/pathogens11060694 (PMC9227307; doi:10.3390/pathogens11060694)
Supplement: Supplementary file 1 [file pathogens-11-00694-s001.zip › Figure S2.pdf]

Figure S2. Alignment of the amino acid sequences included in clade AQP9-like. The amino acids conserved in all the sequences are labelled with asterisks (*); the conservative and semi-conservative substitutions are labelled with colons (:) and periods (.), respectively. The NPA motifs are highlighted in yellow, the residues within the ar/R constriction are highlighted in green, and the aspartic acid downstream the second NPA motif is highlighted in gray. The O-glycosylation sites are marked in blue. The predicted immunogenic peptides from OmAQP90, Om20812 and Om22982 are highlighted in underlined red letters, and their homologous regions in the remaining clade members are highlighted in plain red letters.

```
OmAQP90                MKVYIRSLLLRELINEFIGTMLLILIGDSIMAIIIAGRQEENGHVIGPLGWGTAIFCAVF    60
Om20812                --MKIQSTFVRELINEFLGTFTLIVFGDSVMAIITAGRQEHNAGIIGPVGWGTAIFCAVT    58
Om22982                --MKIQSTFVRELINEFLGTFTLIVFGDSVMAIIIAGRQEHNAGIIGPVGWGTAIFCAVT    58
A0A293N7C6_ORNER       --MQIRNVLARELINEFLGTMILILIGDSIVAVIIAGGQGHNAHVIGPIGWGTAIFCAVF    58
XP_037563606.1         --MKIENLLVRQLINEFLGTLVLVLLGDSVLAIIIAADNESIAAVVGPLGWGTAIYVAVQ    58
XP_037563608.1         --MKIENLLVRQLINEFLGTLVLVLLGDSVLAIIIAADNESIAAVVGPLGWGTAIYVAVQ    58
XP_037563607.1         --MKIENLLVRQLINEFLGTLVLVLLGDSVLAIIIAADNESIAAVVGPLGWGTAIYVAVQ    58
XP_037563609.1         --MKIENLLVRQLINEFLGTLVLVLLGDSVLAIIIAADNESIAAVVGPLGWGTAIYVAVQ    58
XP_037510823.1         --MKIESLLVRQLISEFLGTMILITIGDAIMAIIIAGDNESLAACVGPLGWGVAIYVAVQ    58
XP_037510822.1         --MKIESLLVRQLISEFLGTMILITIGDAIMAIIIAGDNESLAACVGPLGWGVAIYVAVQ    58
XP_037292105.1         --MKIENLLIRQLINEFLGTMILITIGDSIMAIIIAGDNESLAACVGPLGWGVAIYVAVQ    58
XP_037292106.1         --MKIENLLIRQLINEFLGTMILITIGDSIMAIIIAGDNESLAACVGPLGWGVAIYVAVQ    58
AIT69684.1             --MKIENLLIRQLINEFLGTMILITIGDSIMAIIIAGDNESLAACVGPLGWGVAIYVAVQ    58
XP_037292107.1         --MKIENLLIRQLINEFLGTMILITIGDSIMAIIIAGDNESLAACVGPLGWGVAIYVAVQ    58
XP_029845132.1         --MKIESRLIRELINEFIGTMILVLIGDSILAIIIAGDNEPLAPIVGPVGWGTAIFVAVT    58
EEC20079.1             -----------MLARDELIYTLKGLIGDSILAIIIAGDNEPLAPIVGPVGWGTAIFVAVT    49
                                   *  : :       :**:::*:* *. :   .  :**:***.**: **

OmAQP90                VAGGVSAHINPAVTLAFASVKEFPLKKVPLFWLAQYLGAFVGAVLVYVIYRDAINMFDGG    120
Om20812                VSGGVSAHLNPAVTLALASVKKFPIKKVPLFFVAQYTGAFFAAAVVFAIYRDAIDAVDSG    118
Om22982                VSGGVSAHLNPAVTLALASVKKFPIKKVPLFFVAQYTGAFFAAAVVFAIYRDAIDAVDSG    118
A0A293N7C6_ORNER       VAGGVSAHINPAVTLALASAKEFPLKKVPLFWLAQYLGAFVGAVLVYVMYRDVINHFDGG    118
XP_037563606.1         IAGGVTAHLNPAVTLALASVRKFPIAKVPLFFAAQYLGAFVGAALVFVTYKDLIDHFDDG    118
XP_037563608.1         IAGGVTAHLNPAVTLALASVRKFPIAKVPLFFAAQYLGAFVGAALVFVTYKDLIDHFDDG    118
XP_037563607.1         IAGGVTAHLNPAVTLALASVRKFPIAKVPLFFAAQYLGAFVGAALVFVTYKDLIDHFDDG    118
XP_037563609.1         IAGGVTAHLNPAVTLALASVRKFPIAKVPLFFAAQYLGAFVGAALVFVTYKDLIDHFDDG    118
XP_037510823.1         IAGGVSSHLNPAVTLAQASVRKFPIAKVPLYFAAQYLGGFVGAALVFVTYKDAIEHFDQG    118
XP_037510822.1         IAGGVSSHLNPAVTLAQASVRKFPIAKVPLYFAAQYLGGFVGAALVFVTYKDAIEHFDQG    118
XP_037292105.1         ISGGVSSHLNPAVTLAQASVRKFPIAKVPLYFAAQYLGGFVGAALVFATYKDAIEHFDQG    118
XP_037292106.1         ISGGVSSHLNPAVTLAQASVRKFPIAKVPLYFAAQYLGGFVGAALVFATYKDAIEHFDQG    118
AIT69684.1             ISGGVSSHLNPAVTLAQASVRKFPIAKVPLYFAAQYLGGFVGAALVFATYKDAIEHFDQG    118
XP_037292107.1         ISGGVSSHLNPAVTLAQASVRKFPIAKVPLYFAAQYLGGFVGAALVFATYKDAIEHFDQG    118
XP_029845132.1         IAGGVSSHLNPAVTLALASTKKFPINKVPLYFAVQYLGAFVGAALVYLLYHDQIAHFDGG    118
EEC20079.1             IAGGVSSHLNPAVTLALASTKKFPINKVPLYFAVQYLGAFVGAALVYLLYHDQIAHFDGG    109
                       ::***::*:******* **.::**: ****:: .** *.*..*.:*:  *:* *  .* *

OmAQP90                VRSVVGPTGTASIFSTYPREGVSTLTCFFDQVISTAVLTLTVAAIVDERNFA-VPKALVP    179
Om20812                VRSVLGPTGTAPIFATYPREGISTLTCFIDQVVATGCLVLTAEAITDPRNFGGLPKPFYP    178
Om22982                VRSVLGPTGTAPIFATYPREGISTLTCFIDQVVTTGCLVLTAEAITDPRNFGGLPKPFYP    178
A0A293N7C6_ORNER       VRSVVGEKGTAAIFSTYPREEVTTLTCFFDQVISTAVLMLTVAAIVDERNFA-VPKALLP    177
XP_037563606.1         VRHVMGAKATAAIFATYPKPHVSTLTCFIDQVIATGILTLSAEAITDPRNFGGIPVSMHP    178
XP_037563608.1         VRHVMGAKATAAIFATYPKPHVSTLTCFIDQVIATGILTLSAEAITDPRNFGGIPVSMHP    178
XP_037563607.1         VRHVMGAKATAAIFATYPKPHVSTLTCFIDQVIATGILTLSAEAITDPRNFGGIPVSMHP    178
XP_037563609.1         VRHVMGAKATAAIFATYPKPHVSTLTCFIDQVIATGILTLSAEAITDPRNFGGIPVSMHP    178
XP_037510823.1         VRQVTGEKSTAGIFATYPKPHVSTLTCFIDQVIATGIMMVCCEAIGDPRNFGGIPPHIHP    178
XP_037510822.1         VRQVTGEKSTAGIFATYPKPHVSTLTCFIDQVIATGIMMVCCEAIGDPRNFGGIPPHIHP    178
XP_037292105.1         IRQVTGEKATAGIFATYPRPHVSTLTCFIDQVIATGIMMVCVEAIGDTRNFGGIPPHIHP    178
XP_037292106.1         IRQVTGEKATAGIFATYPRPHVSTLTCFIDQVIATGIMMVCVEAIGDTRNFGGIPPHIHP    178
AIT69684.1             IRQVTGEKATAGIFATYPRPHVSTLTCFIDQVIATGIMMVCVEAIGDTRNFGGIPPHIHP    178
XP_037292107.1         IRQVTGEKATAGIFATYPRPHVSTLTCFIDQVIATGIMMVCVEAIGDTRNFGGIPPHIHP    178
XP_029845132.1         VRQITGKHGTAPIFATYPREQISTLTCFFDQLVGTGILMLTAEAITDPRNFGGMPKHLHP    178
EEC20079.1             VRQITGKHGTAPIFATYPREQISTLTCFFDQLVGTGILMLTAEAITDPRNFGGMPKHLHP    169
                       :* : *  .** **:***:  ::*****:**:: *. : :   ** * ***. :*  : *

OmAQP90                LMLGMLIVAEIFAFSYNCMAALNPARDIGPRVFTAVAGWGSEVFSFRNYQWVWVPIFGPH    239
Om20812                LALGLMISAEIFAFSYNCMAPLNPARDLGPRVFTAIAGWGMEVFSVRDYVWMFVPIFAPH    238
Om22982                LALGLMISAEIFAFSYNCMAPLNPARDLGPRVFTAIAGWGMEVFSVRDYVWMFVPIFAPH    238
A0A293N7C6_ORNER       LMLGMLIVAEIFAFSHNCMAALNPARDIGPRIFTAISGWGSQVFTFRNYQWVWVPIFGPH    237
XP_037563606.1         ICLALMIMSLIFSFPYNCMCPLNPARDISPRLFTLIAGWGPETFTLRGGNYVWVPVLGPH    238
XP_037563608.1         ICLALMIMSLIFSFPYNCMCPLNPARDISPRLFTLIAGWGPETFTLRGGNYVWVPVLGPH    238
XP_037563607.1         ICLALMIMSLIFSFPYNCMCPLNPARDISPRLFTLIAGWGPETFTLRGGNYVWVPVLGPH    238
XP_037563609.1         ICLALMIMSLIFSFPYNCMCPLNPARDISPRLFTLIAGWGPETFTLRGGNYVWVPVLGPH    238
XP_037510823.1         ICLGLLIMALIFSFAYNCMCPLNPARDIAPRLFTLMAGWGPETFTLRGGNYVWVPLLGPH    238
```

```
XP_037510822.1         ICLGLLIMALIFSFAYNCMCPLNPARDIAPRLFTLMAGWGPETFTLRGGNYVWVPLLGPH   238
XP_037292105.1         ICLGLMIMAIIFSFAYNCMCPLNPARDISPRLFTLMAGWGPETFTLRGWNYVWVPLLGPH   238
XP_037292106.1         ICLGLMIMAIIFSFAYNCMCPLNPARDISPRLFTLMAGWGPETFTLRGWNYVWVPLLGPH   238
AIT69684.1             ICLGLMIMAIIFSFAYNCMCPLNPARDISPRLFTLMAGWGPETFTLRGWNYVWVPLLGPH   238
XP_037292107.1         ICLGLMIMAIIFSFAYNCMCPLNPARDISPRLFTLMAGWGPETFTLRGWNYVWVPLLGPH   238
XP_029845132.1         LALGFMIMALIFGFSYNCMAPLNPARDIGPRIFTAIAGWGTEVFTYRNWNYIWVPIFGPH   238
EEC20079.1             LALGFMIMALIFGFSYNCMAPLNPARDIGPRIFTAIAGWGTEVFTYRNWNYIWVPIFGPH   229
                       : *.::* : **.* :***. ******:.**:** ::*** :.*: *.  :::**::.**

OmAQP90                IGAIVGVWIYKLCIGDHWPIETTPALKQVLSSSNDKSGPAAEPKETTNI-----------   288
Om20812                VGGVLGAWTYKVCVEDNWPEEEPTVILPVSTSKNSELGPTWL------------------   280
Om22982                VGGVLGAWTYKVCVEDNWPEEEPTVILPVSTSKNSELGTTASAEMNILFS----CDSCV-   293
A0A293N7C6_ORNER       IGAIIGLWIYKLCIRDHWPVQEMTSLKQVVSAASNGDNPGKTVESIVLR--------PAG   289
XP_037563606.1         IGAILGAWLYKVAIADHWPDKPKPTVSTNGESTT-----GRSRPMDCCPM------RKVS   287
XP_037563608.1         IGAILGAWLYKVAIADHWPDKPKPTVST--------------------------------   266
XP_037563607.1         IGAILGAWLYKVAIADHWPDKPKPTVSKELPVPDVAVVDGKKPAAVAAPP------PPAA   292
XP_037563609.1         IGAILGAWLYKVAIADHWPDKPKPTVSTEHLVASPSPG------QL--------------   278
XP_037510823.1         IGAILGVWLYKVAIGDHWPDRPKPAST-N-------------------------------   266
XP_037510822.1         IGAILGVWLYKVAIGDHWPDRPKPAST-NGESTTG-----RPRPTDCWPMR------KVS   286
XP_037292105.1         IGAILGVWLYKVAIGDHWPEKPKPAISTNGEPTTGR---PRPA--DCWP----MRK--VS   287
XP_037292106.1         IGAILGVWLYKVAIGDHWPEKPKPAIST-------------------------------   266
AIT69684.1             IGAILGVWLYKVAIGDHWPEKPKPAISMDHPVAVVV---AKKAPSRTPPALSPSAAPTTS   295
XP_037292107.1         IGAILGVWLYKVAIGDHWPEKPKPAISNEHVVVASP---S---PGQ----LAKEA-----   283
XP_029845132.1         IGAIIGAWIYKVGIGDNFPDDEPKLNGDLERGKVVELQEAAYRPIDDKLNLEAHTNEALK   298
EEC20079.1             IGAIIGAWIYKVGIGDNFPDDEPKLRAAESGVQLVVYQPGAEQPVPEAPAPVP-------   282
                       :*.::* * **: : *::*

OmAQP90                ------------------------------------------   288
Om20812                ------------------------------------------   280
Om22982                ------------------------------------------   293
A0A293N7C6_ORNER       ENR----TEGDQTNTGKI------------------------   303
XP_037563606.1         TSRVVGSPDGREVKDDLVETLYKVDGERLVVELEPSQPQKL-   328
XP_037563608.1         --------DGREVKDDLVETLYKVDGERLVVELEPSQPQKL-   299
XP_037563607.1         AAQAQASPQTA---------------VRQTSDIEAGTPRKSP   319
XP_037563609.1         AKEA--------------------------------------   282
XP_037510823.1         ---------GKETKEDLVETLYKVDGDKMVLELEPAQQHQRL   299
XP_037510822.1         TSRVVGSPNGKETKEDLVETLYKVDGDKMVLELEPAQQHQRL   328
XP_037292105.1         TSRVVGSPDGKETKEDLVETLYKVDGDKMVLELEPTQHQRL-   328
XP_037292106.1         --------DGKETKEDLVETLYKVDGDKMVLELEPTQHQRL-   299
AIT69684.1             TSPQPAAPQATDVNAGS-----------------PAKA----   316
XP_037292107.1         ------------------------------------------   283
XP_029845132.1         L-----------------------------------------   299
EEC20079.1             ------------------------------------------   282
```
