# Supplementary material for: First Data on Ornithodoros moubata Aquaporins: Structural, Phylogenetic and Immunogenic Characterisation as Vaccine Targets
Source: Pathogens. 2022 Jun 16;11(6):694. doi: 10.3390/pathogens11060694 (PMC9227307; doi:10.3390/pathogens11060694)
Supplement: Supplementary file 1 [file pathogens-11-00694-s001.zip › Figure S3.pdf]

Figure S3. Alignment of the amino acid sequences included in clade AQP7-like. The amino acids conserved in all the sequences are labelled with asterisks (*); the conservative and semi-conservative substitutions are labelled with colons (:) and periods (.), respectively. The NPA motifs are highlighted in yellow, the residues within the ar/R constriction are highlighted in green, and the aspartic acid downstream the second NPA motif is highlighted in gray. The predicted immunogenic peptides from OmAQP23 and OmAQP15 are highlighted in underlined red letters and their homologous regions in the remaining clade members are highlighted in plain red letters.

```
OmAQP23                MILDKVKIKSLALRETFAELLGTFVLVLFGTGVLAALKFDRAGNIGYAAGFWGWGLALGM   60
OmAQP15                MILDKVKIKSLALRETFAELLGTYVLVLFGTGVLAALKFDRAGNIGYAAGFWGWGLALGM   60
A0A2R5L6A3_9ACAR       -ILDKVKVKSTAVREMLAELLGTFVLVLFGTGVLAALKFDKTGNIGYAVGFWGWGLALAL   59
A0A293N0K9_ORNER       MITDRIKIRSPAVREMLAELLGTFVLVLFGTGVLASLKFDKVGTIGYAAGFWGWGFALVL   60
A0A293MWN9_ORNER       MITDRIKIRSPAVREMLAELLGTFVLVLFGTGVLASLKFDKVGTIGYAAGFWGWGFALVL   60
A0A1D2AI66_ORNBR       ---DKMKIKSLAVREMLAELLGTFVLVLFGTGVLAALKFDNAGNVGYAAGFWGWGLALAM   57
XP_037563610.1         -MLDSVKIKNAVVREVLAELLGTFVLVLFGNTVLAVVTFDRAPNLGYAAAFWGWALALTL   59
XP_037563613.1         -MLDSVKIKNAVVREVLAELLGTFVLVLFGNTVLAVVTFDRAPNLGYAAAFWGWALALTL   59
XP_037563615.1         -MLDSVKIKNAVVREVLAELLGTFVLVLFGNTVLAVVTFDRAPNLGYAAAFWGWALALTL   59
XP_037510825.1         -MLDSVKIKNALVREVLAELLGTFVLVLFGNTVLAVVTFDRAPNLGYAAAFWGWALALTL   59
XP_037510829.1         -MLDSVKIKNALVREVLAELLGTFVLVLFGNTVLAVVTFDRAPNLGYAAAFWGWALALTL   59
XP_037510831.1         -MLDSVKIKNALVREVLAELLGTFVLVLFGNTVLAVVTFDRAPNLGYAAAFWGWALALTL   59
XP_037510826.1         -MLDSVKIKNALVREVLAELLGTFVLVLFGNTVLAVVTFDRAPNLGYAAAFWGWALALTL   59
CAR66115.1             -MLDSVKIKNALVREVLAELLGTFVLVLFGNTVLAVVAFDRAPNLGYAAAFWGWALALTL   59
XP_037292104.1         -MLDSVKIKNALVREVIAELLGTFVLVLFGNTVLAIVTFDRPPNLGYAAAFWGWALALTL   59
XP_037292102.1         -MLDSVKIKNALVREVIAELLGTFVLVLFGNTVLAIVTFDRPPNLGYAAAFWGWALALTL   59
                          * :*::.  :** :******:******. *** : **.  .:***..****.:** :

OmAQP23                GVLVSGGASGAHLNPSITVALATIGKCQWRKVPHYLVAQYLGAFLGAAVLFLTYKGAFDN   120
OmAQP15                GVLVSGGASGAHLNPSITVALATIGKCQWRKVPHYLVAQYLGAFLGAAVLFLTYKGAFDN   120
A0A2R5L6A3_9ACAR       AVLVAGGASGAHLNPSITIAMATIGKCQWKKVVHYMVAQYLGAFLGAAVLFCTYKNAFDH   119
A0A293N0K9_ORNER       GVLVAGGASGAHLNPSITIALATIGKCEWKKVLHYMVAQYLGAFLAAAVMFCTYKSAFDH   120
A0A293MWN9_ORNER       GVLVAGGASGAHLNPSITIALATIGKCEWKKVLHYMVAQYLGAFLAAAVMFCTYKSAFDH   120
A0A1D2AI66_ORNBR       GVLVSGGASGAHLNPSITVALATIGKCEWRKVLHYLVAQYLGAFFGAAVLFCTYKGAFDH   117
XP_037563610.1         GVLVAGGVSGAHLNPAITVAVTTVGKFPWRKIVPYMVAQYLGAFLASVILFLTYRGALDH   119
XP_037563613.1         GVLVAGGVSGAHLNPAITVAVTTVGKFPWRKIVPYMVAQYLGAFLASVILFLTYRGALDH   119
XP_037563615.1         GVLVAGGVSGAHLNPAITVAVTTVGKFPWRKIVPYMVAQYLGAFLASVILFLTYRGALDH   119
XP_037510825.1         GVLVAGGVSGAHLNPAITVAVTTVGKFPWRKIVPYMVAQYIGAFLASVILFITYRGALDN   119
XP_037510829.1         GVLVAGGVSGAHLNPAITVAVTTVGKFPWRKIVPYMVAQYIGAFLASVILFITYRGALDN   119
XP_037510831.1         GVLVAGGVSGAHLNPAITVAVTTVGKFPWRKIVPYMVAQYIGAFLASVILFITYRGALDN   119
XP_037510826.1         GVLVAGGVSGAHLNPAITVAVTTVGKFPWRKIVPYMVAQYIGAFLASVILFITYRGALDN   119
CAR66115.1             GVLVAGGVSGAHLNPAVTVAVTTVGKFPWRKIVPYMVAQYIGAFLASVILFITYRGALDN   119
XP_037292104.1         GVLVAGGVSGAHLNPAITVAVTTVGKFPWRKIVPYMVAQYIGAFLASVILFITYRGALDN   119
XP_037292102.1         GVLVAGGVSGAHLNPAITVAVTTVGKFPWRKIVPYMVAQYIGAFLASVILFITYRGALDN   119
                       .***:**.*******::*:*::*:**  *:*:  *:****:***:.:.::* **:.*:*:

OmAQP23                YDGGFRATTGVNGTADVFASYPRDFVGTGNCFLDQVVGTGLFVMCIVAITDNHNMAVPQG   180
OmAQP15                YDGGFRATTGVNGTADVFASYPRDFVGTGNCFLDQVVGTGLFVMCIVAITDNHNMAVPQG   180
A0A2R5L6A3_9ACAR       FDGGLRATTGVNATADVFASYPREFVSTGNCFLDQVVGTGLFVMCIVAITDSSNMAVPQG   179
A0A293N0K9_ORNER       YDGGLRATTGVNATADIFASYPRDYVTVANCFLDQVVGTGLFVMCIVAITDSHNMAVPQG   180
A0A293MWN9_ORNER       YDGGLRATTGVNATADIFASYPRDYVTVANCFLDQVVGTGLFVMCIVAITDSHNMAVPQG   180
A0A1D2AI66_ORNBR       FDGGNRTTTGATATADIFASYPREFVTTGNCFLDQVVGTGLFVMCIIAITDNSNMSVAQG   177
XP_037563610.1         YDGGVRAVTGENATAGIFASYPQEFVSTGNGLVDQIVGTALLVLVILAITDSRNMAVPQG   179
XP_037563613.1         YDGGVRAVTGENATAGIFASYPQEFVSTGNGLVDQIVGTALLVLVILAITDSRNMAVPQG   179
XP_037563615.1         YDGGVRAVTGENATAGIFASYPQEFVSTGNGLVDQIVGTALLVLVILAITDSRNMAVPQG   179
XP_037510825.1         YDGGVRAVTGSNATAGIFASYPQEFVSTGNGLVDQIVGTALLVLAILAITDSRNMAVPQG   179
XP_037510829.1         YDGGVRAVTGSNATAGIFASYPQEFVSTGNGLVDQIVGTALLVLAILAITDSRNMAVPQG   179
XP_037510831.1         YDGGVRAVTGSNATAGIFASYPQEFVSTGNGLVDQIVGTALLVLAILAITDSRNMAVPQG   179
XP_037510826.1         YDGGVRAVTGSNATAGIFASYPQEFVSTGNGLVDQIVGTALLVLAILAITDSRNMAVPQG   179
CAR66115.1             YDGGVRAVTGSNATAGIFASYPQEFVSTGNGLVDQIVGTALLVLAILAITDSRNMAVPQG   179
XP_037292104.1         YDGGVRAVTGANATAGIFASYPQEFVSTGNGLVDQIVGTALLVLAILAITDSRNMAVPQG   179
XP_037292102.1         YDGGVRAVTGANATAGIFASYPQEFVSTGNGLVDQIVGTALLVLAILAITDSRNMAVPQG   179
                       :*** *:.** ..**.:*****:::* ..* ::**:***.*:*: *:****. **:* **

OmAQP23                LQPLLIGLSLATTALCFGYNCGAPLNPARDLGPRIFTAMAGWGTEVFSFRDYNWFWVPVV   240
OmAQP15                LQPLLIGLSLATTALCFGYNCGAPLNPARDLGPRIFTAMAGWGTEVFSFRDYNWFWVPVV   240
A0A2R5L6A3_9ACAR       LQPLLIGISLATTALCFGYNCGAPLNPARDLGPRIFTAMAGWGTEVFSFRDYNWFWVPVV   239
A0A293N0K9_ORNER       LQPLLIGIALATTALCFGYNCGAPLNPARDLGPRVFTAMAGWGTEVFSFRDYNWFWVPIV   240
A0A293MWN9_ORNER       LQPLLIGIALATTALCFGYNCGAPLNPARDLGPRVFTAMAGWGTEVFSFRDYNWFWVPIV   240
A0A1D2AI66_ORNBR       LQPLLIGLSLATTALCFGYNCGAPLNPARDLGPRIFTSMAGWGTEVFSFRDYNWFWVPIV   237
XP_037563610.1         IQPLFIGLALGALILCFGYNCGAPLNPARDLAPRVFTAMAGWGVEVFSYRDYNWFWVPIV   239
XP_037563613.1         IQPLFIGLALGALILCFGYNCGAPLNPARDLAPRVFTAMAGWGVEVFSYRDYNWFWVPIV   239
XP_037563615.1         IQPLFIGLALGALILCFGYNCGAPLNPARDLAPRVFTAMAGWGVEVFSYRDYNWFWVPIV   239
XP_037510825.1         VQPLFIGLALGALILCFGYNCGAPLNPARDLGPRVFTAMAGWGVEVFSYRDYNWFWVPIV   239
```

```
XP_037510829.1        VQPLFIGLALGALILCFGYNCGAPLNPARDLGPRVFTAMAGWGVEVFSYRDYNWFWVPIV    239
XP_037510831.1        VQPLFIGLALGALILCFGYNCGAPLNPARDLGPRVFTAMAGWGVEVFSYRDYNWFWVPIV    239
XP_037510826.1        VQPLFIGLALGALILCFGYNCGAPLNPARDLGPRVFTAMAGWGVEVFSYRDYNWFWVPIV    239
CAR66115.1            VQPLFIGLALGALILCFGYNCGAPLNPARDLGPRVFTAMAGWGVEVFSYRDYNWFWVPIV    239
XP_037292104.1        VQPLFIGLALGAIILCFGYNCGAPLNPARDLGPRVFTAMAGWGVEVFSYRDYNWFWVPIV    239
XP_037292102.1        VQPLFIGLALGAIILCFGYNCGAPLNPARDLGPRVFTAMAGWGVEVFSYRDYNWFWVPIV    239
                      :***:**::*.:  *****************.**:**:*****.****:*********:*

OmAQP23               APHIGAIVGAWLYRLLVGLHWPQDSYDMEQRNRAKDGGY----SLE--------------    282
OmAQP15               APHIGAIVGTWLYRLLVGLHWPQDSYDMEQRNRAKDEYM----PIGGYDAAPKEQQSASS    296
A0A2R5L6A3_9ACAR      APHIGAVVGTWLYKLLVGLHWPQDSYDMDQGRRDKDGGY----TLERRN-----------    284
A0A293N0K9_ORNER      APHIGAVVGTWLYRLLVGLHWPQDSYDMEQGRRSKDGGY----TLESRN-----------    285
A0A293MWN9_ORNER      APHIGAVVGTWLYRLLVGLHWPQDSYDMEQGRRSKDEYL----PVRGDDGAPPERGLDLS    296
A0A1D2AI66_ORNBR      APHLGAIIGTWLYKLMVGLHWPQDSYDMEQGGRGKDGGY----SLEGRN-----------    282
XP_037563610.1        GPHIGAIVGAWLYMLAVELHWPGSSYDMDSGNAVSAKDVENVIQMRGIKQCEIKN-----    294
XP_037563613.1        GPHIGAIVGAWLYMLAVELHWPGSSYDMDSGNAVSAKDDAYGQ--DGVRM----------    287
XP_037563615.1        GPHIGAIVGAWLYMLAVELHWPGSSYDMDSGNAVSAKDETVST--M--------------    283
XP_037510825.1        GPHIGAIVGAWLYTLAVELHWPGSSYDMDSGNTVSAKDVENVIQMRGIKQCELKN-----    294
XP_037510829.1        GPHIGAIVGAWLYTLAVELHWPGSSYDMDSGNTVSAKDDGIGPDSVRM------------    287
XP_037510831.1        GPHIGAIVGAWLYTLAVELHWPGSSYDMDSGNTVSAKDEPVSSM----------------    283
XP_037510826.1        GPHIGAIVGAWLYTLAVELHWPGSSYDMDSGNTVSAKDAPRFQLANQNEAIF--------    291
CAR66115.1            GPHIGAIVGAWLYTLAVELHWPGSSYDMDSGNTVSAKDAPRFQLANQNEAIF--------    291
XP_037292104.1        GPHIGAIVGAWLYTLAVELHWPGSSYDMDSGNAVSAKDEPVSSK----------------    283
XP_037292102.1        GPHIGAIVGAWLYTLAVELHWPGSSYDMDSGNAVSAKDDGIGQDSVRM------------    287
                      .**:**::*:*** * * **** .****:.    .

OmAQP23               -----------------------   282
OmAQP15               PNEKQSKV---------------   304
A0A2R5L6A3_9ACAR      -----------------------   284
A0A293N0K9_ORNER      -----------------------   285
A0A293MWN9_ORNER      SSTHEKRPQSGTGGSGNGTNRRS   319
A0A1D2AI66_ORNBR      -----------------------   282
XP_037563610.1        -----------------------   294
XP_037563613.1        -----------------------   287
XP_037563615.1        -----------------------   283
XP_037510825.1        -----------------------   294
XP_037510829.1        -----------------------   287
XP_037510831.1        -----------------------   283
XP_037510826.1        -----------------------   291
CAR66115.1            -----------------------   291
XP_037292104.1        -----------------------   283
XP_037292102.1        -----------------------   287
```
