# Supplementary material for: First Data on Ornithodoros moubata Aquaporins: Structural, Phylogenetic and Immunogenic Characterisation as Vaccine Targets
Source: Pathogens. 2022 Jun 16;11(6):694. doi: 10.3390/pathogens11060694 (PMC9227307; doi:10.3390/pathogens11060694)
Supplement: Supplementary file 1 [file pathogens-11-00694-s001.zip › Figure S4.pdf]

Figure S4. Alignment of the amino acid sequences included in clade AQP7/AQP9/AQP3. The amino acids conserved in all the sequences are labelled with asterisks (*); the conservative and semi-conservative substitutions are labelled with colons (:) and periods (.), respectively The NPA motifs are highlighted in yellow, the residues within the ar/R constriction are highlighted in green, and the aspartic acid downstream the second NPA motif is highlighted in gray. The predicted immunogenic peptides from OmAQP97 are highlighted in underlined red letters and their homologous regions in the remaining clade members are highlighted in plain red letters.

```
OmAQP97                   ---MANPPFPFMKRFVIKSPVLREFLAEFFGTFILYIFGGASFAHYIFSGQKDVLAVTFC     57
A0A293N0E3_ORNER          ---MANPPYPFLKRFVIRSPAVREFLAEFFGTFILYIFGGASFAHYIFGNQKDVLAVTFC     57
A0A2R5L6Q7_9ACAR          ---MANPPYPFLKRFVIKSPALREFLAEFFGTFILYIFGGASFAHYIFGNQKDVLAVTFC     57
XP_029845129.1            ---MGNPPFPFFKKFVTTNQAVREFLAEFFGTFILYIFGGASFAHYLFTDNKDVFAVTFC     57
XP_037563616.1            MASAGNPPFPFLRKLVTKNEAVKEFLAEFFGTFILYMFGGASFAHFLFTDSKDVFAVTFC     60
XP_037292097.1            MASAGNPPFPFLRKFVTKNEAVKEFLAEFLGTFILYMFGGASFAHFLFTDQKDVFAVTFC     60
XP_037292096.1            MASAGNPPFPFLRKFVTKNEAVKEFLAEFLGTFILYMFGGASFAHFLFTDQKDVFAVTFC     60
XP_037509276.1            --------------------------------MEVKMFGGASFAHFLFTDQKDVFAVTFC     28
XP_037510820.1            MASAGNPPFPFLRKFVTKNEAVKEFLAEFLGTFILYMFGGASFAHFLFTDQKDVFAVTFC     60
XP_037510819.1            MASAGNPPFPFLRKFVTKNEAVKEFLAEFLGTFILYMFGGASFAHFLFTDQKDVFAVTFC     60
CAX48963.1                MASAGNPPFPFLRKFVTKNEAVKEFLAEFLGTFILYMFGGASFAHYLFTAQKDVFAATFC     60
                                                          : : :********::*  .***:*.***

OmAQP97                   WGMGVMLGIQTGAGVSGGHVNPVVTTPFASVGRLPWKKVPHYLLGQHLGAFLASAILYFN     117
A0A293N0E3_ORNER          WGMGVMLGIQTGAGVSGGHVNPVVTTSFASVGRLPWRKVPHYILGQHLGAFLASAILYFN     117
A0A2R5L6Q7_9ACAR          WGMGVMLGIQTGAGVSGGHVNPVVTTSFASVGRLPWRKVPHYILGQHLGAFVASAILYFN     117
XP_029845129.1            WGIGVMLGIQTGAGVSGGHVNPIVTTSFASVGKLPWRKVPHYLLGQHLGAFLAAAILYFN     117
XP_037563616.1            WGIGVMLGIQTGAGVSGGHVNPIVTTSFASIGKLPWRKVPHYMLGQHLGAFLASAILYFN     120
XP_037292097.1            WGIGVMLGIQTGAGVSGGHVNPIVTTSFASVGKLPWRKVPHYILGQHLGAFLASAILYFN     120
XP_037292096.1            WGIGVMLGIQTGAGVSGGHVNPIVTTSFASVGKLPWRKVPHYILGQHLGAFLASAILYFN     120
XP_037509276.1            WGIGVMLGIQTGAGVSGGHVNPIVTTSFASVGKLPWRKVPHYILGQHLGAFLASAILYFN     88
XP_037510820.1            WGIGVMLGIQTGAGVSGGHVNPIVTTSFASVGKLPWRKVPHYILGQHLGAFLASAILYFN     120
XP_037510819.1            WGIGVMLGIQTGAGVSGGHVNPIVTTSFASVGKLPWRKVPHYILGQHLGAFLASAILYFN     120
CAX48963.1                WGIGVMLGIQTGAGVSGGHVNPIVTTSFASVGKLPWRKVPHYILGQHLGAFLASAILYFN     120
                          **:*******************:*** ***:*:***:*****:********:*:******

OmAQP97                   YIDALDHYDGGERQIFGDRGTGILLTTFPNEHVHLSICIFDTIICSGLLMFTIMVIIDNK     177
A0A293N0E3_ORNER          YVDVLDHYDGGTRQIFGVNGTGILLTTFPNEHVHLSICIFDTIICSALLMFTIMVIIDNK     177
A0A2R5L6Q7_9ACAR          YIDVLDHYDGGERQIFGNKGTGILLTTFPNENVHLSICIFDTIICSALLMFTIMVIIDNK     177
XP_029845129.1            YIDLLDKFDGGARQIFGTNGTGILLSTYPHPDVHLSVCVFDTIICSGLLMFTIMVILDDR     177
XP_037563616.1            YIDLLDSVDGGERQIFGKNGTGILLTTYPNDNVHLSVCVFDTIICSALLMFTIMVILDDR     180
XP_037292097.1            YIDLLDSVDGGERQIFGKNGTGILLTTYPNDNVHLSVCVFDTIICSGLLMFTIMVILDDR     180
XP_037292096.1            YIDLLDSVDGGERQIFGKNGTGILLTTYPNDNVHLSVCVFDTIICSGLLMFTIMVILDDR     180
XP_037509276.1            YIDLLDSVDGGERQIFGKNGTGVLLTTYPNDHVHLSVCVFDTIICSGLLMFTIMVILDDR     148
XP_037510820.1            YIDLLDSVDGGERQIFGKNGTGVLLTTYPNDHVHLSVCVFDTIICSGLLMFTIMVILDDR     180
XP_037510819.1            YIDLLDSVDGGERQIFGKNGTGVLLTTYPNDHVHLSVCVFDTIICSGLLMFTIMVILDDR     180
CAX48963.1                YIDLLDSVDGGERQIFGKNGTGVLLTTYPNDHVHLSVCVFDTIICSGLLMFTIMVILDDR     180
                          *:* **  *** ***** .***:**:*:*: .****:*:*******.*********:*::

OmAQP97                   NANTTSGMHAFLIGIMVMALCWAFGTNCMAAVNPARDFPPRVLASIVGYGPEVFTYRHFW     237
A0A293N0E3_ORNER          NANTTAGMHAFLIGIMVIALCWAFGTNCMAAINPARDFPPRVLASMVGYGPEVFTYRHFW     237
A0A2R5L6Q7_9ACAR          NANTTAGMHAFLIGIMVIALCWAFGTNCMAAINPARDFPPRVLASMVGYGPEVFTFRHFW     237
XP_029845129.1            NANTTKGMHAFLIGIMVVALCWAFGSNCMAAVNPARDFPPRVLSALVGYGAETFSYRQFW     237
XP_037563616.1            NANTTKGMHAFLIGIMVVALCWAYGANCMAAVNPARDFPARVLSALVGYGAETFTFRNFW     240
XP_037292097.1            NANTTKGMHAFLIGIMVVALCWAYGANCMGAVNPARDFPARVLSAIVGYGAESFTFRNFW     240
XP_037292096.1            NANTTKGMHAFLIGIMVVALCWAYGANCMGAVNPARDFPARVLSAIVGYGAESFTFRNFW     240
XP_037509276.1            NANTTKGMHAFLIGIMVVALCWAYGANCMAAVNPARDFPARVLSALVGYGAESFMFRNFW     208
XP_037510820.1            NANTTKGMHAFLIGIMVVALCWAYGANCMAAVNPARDFPARVLSALVGYGAESFTFRNFW     240
XP_037510819.1            NANTTKGMHAFLIGIMVVALCWAYGANCMAAVNPARDFPARVLSALVGYGAESFTFRNFW     240
CAX48963.1                NANTTKGMHAFLIGIMVVALCWAYGANCMAAVNPARDFPARVLSALVGYGAESFMFRNFW     240
                          ***** ***********:*****:*:***.*:******* ***:::**** * * :*:**

OmAQP97                   WIPMFIPHVGGLIGTWLYVLAIEMHIVPERVRDDRTEREMTNLLPLPPRTEKTGV-----     292
A0A293N0E3_ORNER          WIPMFIPHVGGLIGTWLYVLAIEMHIVPERARDDRTEREMTNLLPLPPRTEKPAI-----     292
A0A2R5L6Q7_9ACAR          WIPMFIPHVGGLIGTWLYVLAIEMHIRPERLHDDRAEREMTNLLPLPPRTEKSAA-----     292
XP_029845129.1            WIPLFVPHLGGFIGTWLYVLFVGMHIPKPRRDGD--EKEVVNLLPLAPRADKTSP-----     290
XP_037563616.1            WIPMFVPHLGGLIGTWLYVLGVGMHISKPQSK-D--GPEGVNLLPLAPRADKTVA-----     292
XP_037292097.1            WIPMFVPHLGGLIGTWLYVLCIGMHISKPQSR-D--GPEGVNLLPLAPRADKTAA-----     292
XP_037292096.1            WIPMFVPHLGGLIGTWLYVLCIGMHISKPQSR-D--GPEGVNLLPLAPRAGESTGNTGVY     297
XP_037509276.1            WIPMFVPHLGGLIGTWLYVLCIGMHISKPQQK-D--GPEGVNLLPLAPRADKAVA-----     260
XP_037510820.1            WIPMFVPHLGGLIGTWLYVLCIGMHISKPQQK-D--GPEGVNLLPLAPRADKAVA-----     292
XP_037510819.1            WIPMFVPHLGGLIGTWLYVLCIGMHISKPQQK-D--GPEGVNLLPLAPRAGESTGNTGVY     297
CAX48963.1                WIPMFVPHLGGLIGTWLYVLCIGMHISKPQQK-D--GPEGVNLLPLAPRAGESTGNTGVY     297
                          ***:*:**:**:******** : ***   :   *    * .***** **: :
```

```
OmAQP97               -------------------------------------     292
A0A293N0E3_ORNER      -------------------------------------     292
A0A2R5L6Q7_9ACAR      -------------------------------------     292
XP_029845129.1        -------------------------------------     290
XP_037563616.1        -------------------------------------     292
XP_037292097.1        -------------------------------------     292
XP_037292096.1        TVGLHSAVYHIADNKGTNANIASPGVPAGNVSEEKVK     334
XP_037509276.1        -------------------------------------     260
XP_037510820.1        -------------------------------------     292
XP_037510819.1        TVGLRSAVYHITDDRGTHAIVASPGVPAANIIEEKIK     334
CAX48963.1            TVGLRSAVYHITDDRGTHANVASPGVPAANIIEEKIK     334
```
