# Supplementary material for: First Data on Ornithodoros moubata Aquaporins: Structural, Phylogenetic and Immunogenic Characterisation as Vaccine Targets
Source: Pathogens. 2022 Jun 16;11(6):694. doi: 10.3390/pathogens11060694 (PMC9227307; doi:10.3390/pathogens11060694)
Supplement: Supplementary file 1 [file pathogens-11-00694-s001.zip › Figure S5.pdf]

Figure S5. Alignment of the amino acid sequences included in clade AQPAe.a. The amino acids conserved in all the sequences are labelled with asterisks (*); the conservative and semi-conservative substitutions are labelled with colons (:) and periods (.), respectively. The NPA motifs are highlighted in yellow and the residues within the ar/R constriction are highlighted in green. The predicted immunogenic peptides from OmAQP91 are highlighted in underlined red letters and their homologous regions in the remaining clade members are highlighted in plain red letters.

```
OmAQP91                MGRVRQFRSIFGIDELSATSTLWKSMIAEYIGTAMLVFIACGTCTNWGRGG----EPSIA    56
A0A293MYA1_ORNER       MGRVRQFRSVFGIDELSATSTLWKAMIAEYIGTAVLVFIACGTCTNWGRPG----EPSIT    56
XP_029833586.1         MGRVRQFRNLCGIDEVSASSTLWKSMVAEYIGTAVIVLIGCGSCINWGSPTKPLGEATMV    60
XP_037582105.1         MGRVRQFRSFCGIDEVSATSSLWKSMVAEYIGTAVIVLIGCGSCINWGSPEKPQGEATMV    60
XP_037502974.1         MGRVRQFRSFCGIDEVSATSSLWKSMVAEYIGTAVIVLIGCGSCINWGSPEKPQGEATMV    60
XP_037283568.1         MGRVRQFRSFCGIDEVSATSSLWKSMVAEYIGTAVIVLIGCGSCINWGSPEKPQGEATMV    60
                       ********.. ****:**:*:***:*:*******::*:*.**:* ***       * ::.

OmAQP91                QIALSFAFIVATMAQALGHVSGAHINPAVTMGMLCVGHISILRAFFYICSQLIGGITGAA    116
A0A293MYA1_ORNER       QIALSFAFIVATMAQALGHVSGAHINPAVTMGMLCVGHVSILRAFFYICSQLIGGITGAA    116
XP_029833586.1         QIAIGFGITVATMAQAVGHVSGAHLNPAVTMGMLFVGKVSILRSFFYICSQLIGGITGAA    120
XP_037582105.1         QIAIGFGVTVATMVQALGHVSGGHLNPAVTIGMLFVGRVSLLRSFFYLCSQLIGGITGAA    120
XP_037502974.1         QIAIGFGITVASMVQALGHVSGGHLNPAVTIGMLFVGRVSLLRSFFYLCSQLIGGITGAA    120
XP_037283568.1         QIAIGFGITVASMVQALGHVSGGHLNPAVTIGMLFVGRVSLLRSFFYLCSQLIGGITGAA    120
                       ***:.*.. **:*.**:*****.*:*****:*** **::*:**:***:************

OmAQP91                VLRAVTPEERQGLLGGTALSEGVTPFQGVVVELVITFILVLTVFAVCDQNRLDVLGSPPL    176
A0A293MYA1_ORNER       ILRAVTPEDRQGALGGTKLSDGVTPFQGVVVELMITFILVLTVFAVCDQNRLDILGSAPL    176
XP_029833586.1         ILRAVTPDARHGALGGTGLAEGVTPLMGLAVEVCITFILVLTVFSVCDTNRLDVQGSAPL    180
XP_037582105.1         ILRAVTPEARHGALGGTSLAEGVTPLMGLGVEACITFILVLTVFGVCDTNRLDVQGSAPL    180
XP_037502974.1         ILRAVTPEARHGALGGTSLAEGVTPLMGLGVEACITFILVLTVFGVCDANRLDVQGSAPL    180
XP_037283568.1         ILRAVTPEARHGALGGTSLAEGVTPLMGLGVEACITFILVLTVFGVCDANRLDVQGSAPL    180
                       :******: *:* **** *::****: *: **  **********.*** ****: ** **

OmAQP91                AIGLAVGAGHLMVIGYTGASMNTARTFGPAVISGAFDDHWVYWLGPIGGGILAGFVYQYV    236
A0A293MYA1_ORNER       AIGLSVGAGHLMAIGYTGASMNTARTFGPAVISGAFDDHWVYWLGPIGGGILAGFVYQYV    236
XP_029833586.1         AIGLSVTTCHVFAVRYTGAGMNVARSFGPAVMSGIFDDHWVFWVGPILGGIIAAVIYENV    240
XP_037582105.1         AIGLSVTTCHVFAVRYTGAGMNVARSFGPAVMSGMFDDHWVFWLGPILGGVTAAVVYENV    240
XP_037502974.1         AIGLSVTTCHVFAVRYTGAGMNVARSFGPAVMSGMFDDHWVFWLGPILGGVIAAVVYENV    240
XP_037283568.1         AIGLSVTTCHVFAVRYTGAGMNVARSFGPAVMSGMFDDHWVFWLGPILGGVIAAVVYENV    240
                       ****:* : *:::.:****.**.**:*****:** ******:*:*** **: *..:*: *

OmAQP91                LSAPPISFEALDKAQLACLARFHNKEAVEDRTTAI         271
A0A293MYA1_ORNER       FSAPAITSEALDKAQLASLAKFNNKEVVEDRTTSI         271
XP_029833586.1         FQAPPITTEELDKAQITAMAKLANKEVVADRTTSI         275
XP_037582105.1         FQAPPITSEDLDKAQITVMAKLANKEVVADRTTSI         275
XP_037502974.1         FQAPPITTEDLDKAQITVMAKLANKEVVADRTTSI         275
XP_037283568.1         FQAPPITSEDLDKAQIMAMAKLTNKEVVADRTTSI         275
                       :.** *: * *****:  :*:: ***.* ****:*
```
