# Supplementary material for: First Data on Ornithodoros moubata Aquaporins: Structural, Phylogenetic and Immunogenic Characterisation as Vaccine Targets
Source: Pathogens. 2022 Jun 16;11(6):694. doi: 10.3390/pathogens11060694 (PMC9227307; doi:10.3390/pathogens11060694)

**Figure S9.** 3D models for OmAQP15. (a) and (b) Side and top view, respectively, of the monomer cartoon showing the position of the two NPA motifs (red spheres) and the aromatic arginine (ar/R) constriction (orange spheres); (c) and (d) side and top view of the tetramer cartoon; (e) monomer mesh and surface model showing the exposure of the 3 antigenic peptides on the extracellular surface of the molecule: OmAQP15_A yellow, OmAQP15_C orange, and OmAQP15_E cyan. (f) side and top view of tetramer surface model showing peptide exposure.

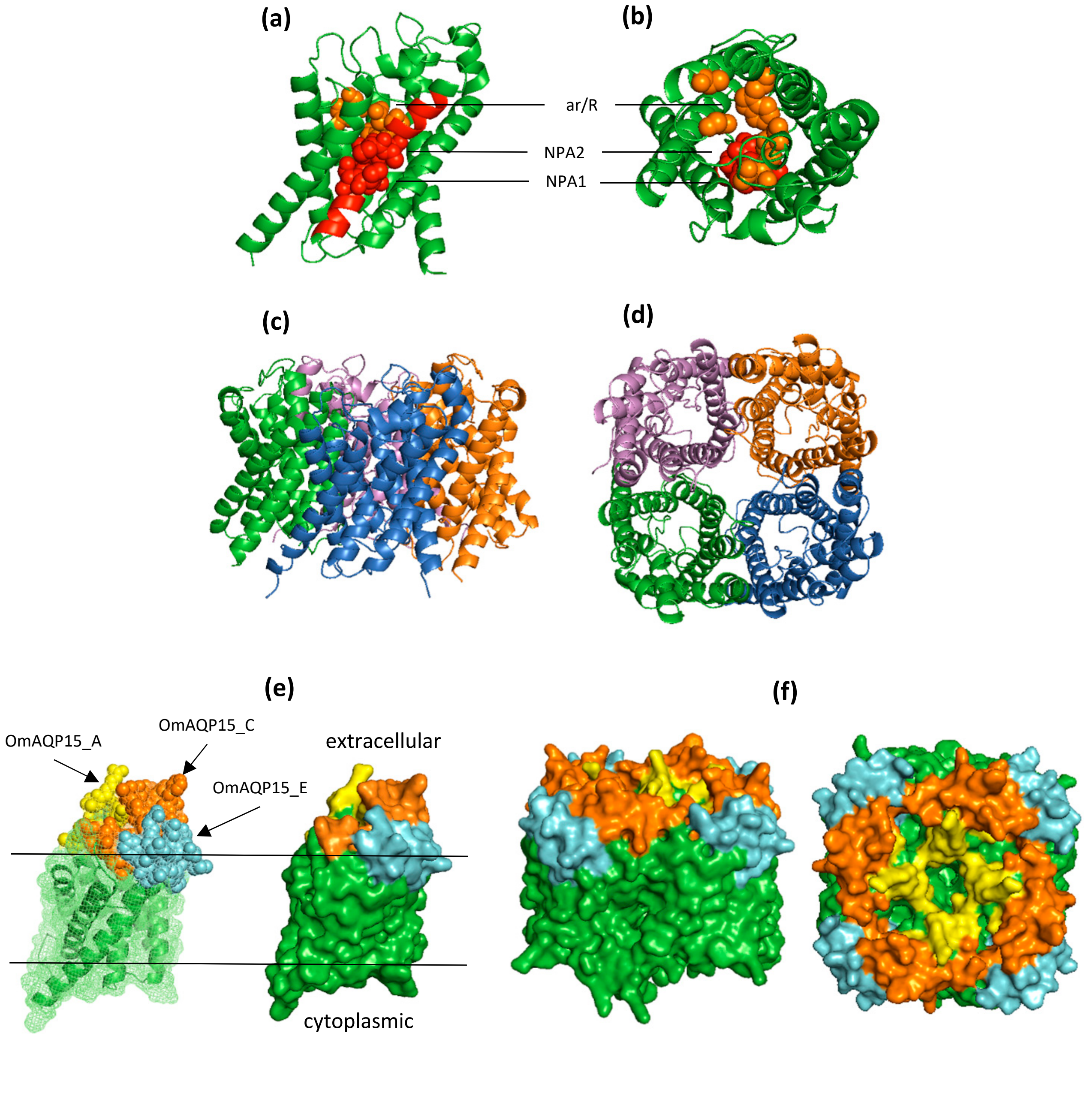

Supplement: Supplementary file 1 [file pathogens-11-00694-s001.zip › Figure S9.pdf]
